# Allantodapsone is a Pan-Inhibitor of Staphylococcus aureus Adhesion to Fibrinogen, Loricrin, and Cytokeratin 10

**DOI:** 10.1128/spectrum.01175-21

**Published:** 2022-06-01

**Authors:** Filippo Prencipe, Aishah Alsibaee, Zainab Khaddem, Padraig Norton, Aisling M. Towell, Afnan F. M. Ali, Gerard Reid, Orla M. Fleury, Timothy J. Foster, Joan A. Geoghegan, Isabel Rozas, Marian P. Brennan

**Affiliations:** a School of Chemistry, Trinity Biomedical Sciences Institute, Trinity College Dublin, Dublin, Ireland; b School of Pharmacy and Biomedical Sciences, RCSI University of Medicine and Health Sciences, Dublin, Ireland; c Department of Microbiology, Moyne Institute of Preventive Medicine, School of Genetics and Microbiology, Trinity College Dublin, Dublin, Ireland; d Institute of Microbiology and Infection, University of Birmingham, Edgbaston, Birmingham, United Kingdom; Riverside University Health System, Medical Center—University of California

**Keywords:** ClfA, ClfB, MRSA, MSSA, *Staphylococcus aureus*, allantodapsone, atopic dermatitis, clumping factors, cytokeratin 10, loricrin, nasal colonization, wound infection

## Abstract

Staphylococcus aureus infections have become a major challenge in health care due to increasing antibiotic resistance. We aimed to design small molecule inhibitors of S. aureus surface proteins to be developed as colonization inhibitors. We identified allantodapsone in an initial screen searching for inhibitors of clumping factors A and B (ClfA and ClfB). We used microbial adhesion assays to investigate the effect of allantodapsone on extracellular matrix protein interactions. Allantodapsone inhibited S. aureus Newman adhesion to fibrinogen with an IC_50_ of 21.3 μM (95% CI 4.5-102 μM), minimum adhesion inhibitory concentration (MAIC) of 100 μM (40.2 μg/mL). Additionally, allantodapsone inhibited adhesion of Lactococcus lactis strains exogenously expressing the clumping factors to fibrinogen (L. lactis ClfA, IC_50_ of 3.8 μM [95% CI 1.0–14.3 μM], MAIC 10 μM, 4.0 μg/mL; and L. lactis ClfB, IC_50_ of 11.0 μM [95% CI 0.9–13.6 μM], MAIC 33 μM, 13.3 μg/mL), indicating specific inhibition. Furthermore, the dapsone and alloxan fragments of allantodapsone did not have any inhibitory effect. Adhesion of S. aureus Newman to L2v loricrin is dependent on the expression of ClfB. Allantodapsone caused a dose dependent inhibition of S. aureus adhesion to the L2v loricrin fragment, with full inhibition at 40 μM (OD_600_ 0.11 ± 0.01). Furthermore, recombinant ClfB protein binding to L2v loricrin was inhibited by allantodapsone (*P < *0.0001). Allantodapsone also demonstrated dose dependent inhibition of S. aureus Newman adhesion to cytokeratin 10 (CK10). Allantodapsone is the first small molecule inhibitor of the S. aureus clumping factors with potential for development as a colonization inhibitor.

**IMPORTANCE** S. aureus colonization of the nares and the skin provide a reservoir of bacteria that can be transferred to wounds that can ultimately result in systemic infections. Antibiotic resistance can make these infections difficult to treat with significant associated morbidity and mortality. We have identified and characterized a first-in-class small molecule inhibitor of the S. aureus clumping factors A and B, which has the potential to be developed further as a colonization inhibitor.

## INTRODUCTION

Antibiotic resistance is one of the greatest challenges to health care, and S. aureus infections are increasingly becoming a problem in the clinic. S. aureus is responsible for serious infections such as infective endocarditis, pneumonia, infections of joints, indwelling devices, and sepsis. It is also a major cause for abscesses, impetigo, and surgical wound infections, which often lead to severe complications for patients. Methicillin resistant S. aureus (MRSA) is now widespread both in the community and in patient populations. Even though this can be treated relatively successfully with vancomycin or daptomycin, mortality rates associated with infection are still extremely high. Susceptibility to vancomycin has also decreased in some strains, with a rise in vancomycin intermediate S. aureus (VISA) strains. Although daptomycin can be used for vancomycin failures, daptomycin resistance has also developed in some S. aureus strains (1). Increasing antibiotic resistance and multidrug resistant strains provide a strong case for the development of novel treatments for S. aureus infection targeting novel mechanisms of action. Nasal colonization with MRSA is a source of transmission within the hospital setting. The antibiotic mupirocin is used for nasal decolonization of MRSA; however, mupirocin resistant strains have emerged.

The microbial surface components recognizing adhesive matrix molecules (MSCRAMMs) are a well characterized set of surface proteins that are involved in host–pathogen interactions including wound and nasal colonization, as well as interactions with platelets. The ubiquitous clumping factors (ClfA and ClfB) have been identified as virulence factors present in both methicillin sensitive and methicillin resistant strains and thus are considered essential for S. aureus colonization and infection (2). These clumping factors are compelling drug targets because of their surface expression and their ability to interact with multiple ligands. They can adhere to exposed fibrinogen in wounds when the extracellular matrix is exposed. Furthermore, they clump bacteria together in the presence of soluble fibrinogen and can lead to platelet activation and aggregation (3, 4), which is responsible for the thrombotic complications associated with systemic infections. ClfA binds to the C-terminus of the γ-chain of fibrinogen (5). ClfB binds to the C-terminus of the α-chain of fibrinogen (6, 7), as well as to cytokeratin 10 (CK10) (8, 9), cytokeratin 8 (10), and loricrin (11, 12). The interaction with loricrin has been shown to be key in nasal colonization (11). While these two clumping factors have different peptide ligands, they have extremely similar three-dimensional structures in their ligand binding domains, which have a groove that runs along between the N2 and N3 subunits of the A-domain. Co-crystal structures of ClfA complexed with fibrinogen (13) and ClfB with fibrinogen, CK10, and dermokine have been solved (14, 15) allowing for detailed characterization of these interactions.

The objective of this project was to employ *in silico* methods to develop molecules that could potentially fit into the ligand binding trenches of both ClfA and ClfB. Such a molecule might be the prototype of a pan-inhibitor of bacterial adhesion for inhibiting colonization of the nares and surgical wounds. Allantodapsone was identified and its ability to inhibit bacterial adhesion to immobilized ligands was tested. The activity of the molecule was explored using substructure fragments and synthesized derivatives.

Here we present a novel inhibitor of S. aureus adhesion, allantodapsone, that inhibits adhesion to fibrinogen, loricrin, and cytokeratin 10. This inhibitor targets bacterial adhesion through inhibition of the ligand binding activity of ClfA and ClfB. This is the first reported small molecule inhibitor for any MSCRAMM and thus has the potential to be developed as an inhibitor of nasal or skin colonization or for the treatment of wound infections.

## RESULTS

### Inhibition of S. aureus adhesion to ligands by allantodapsone.

**FIG 1:**
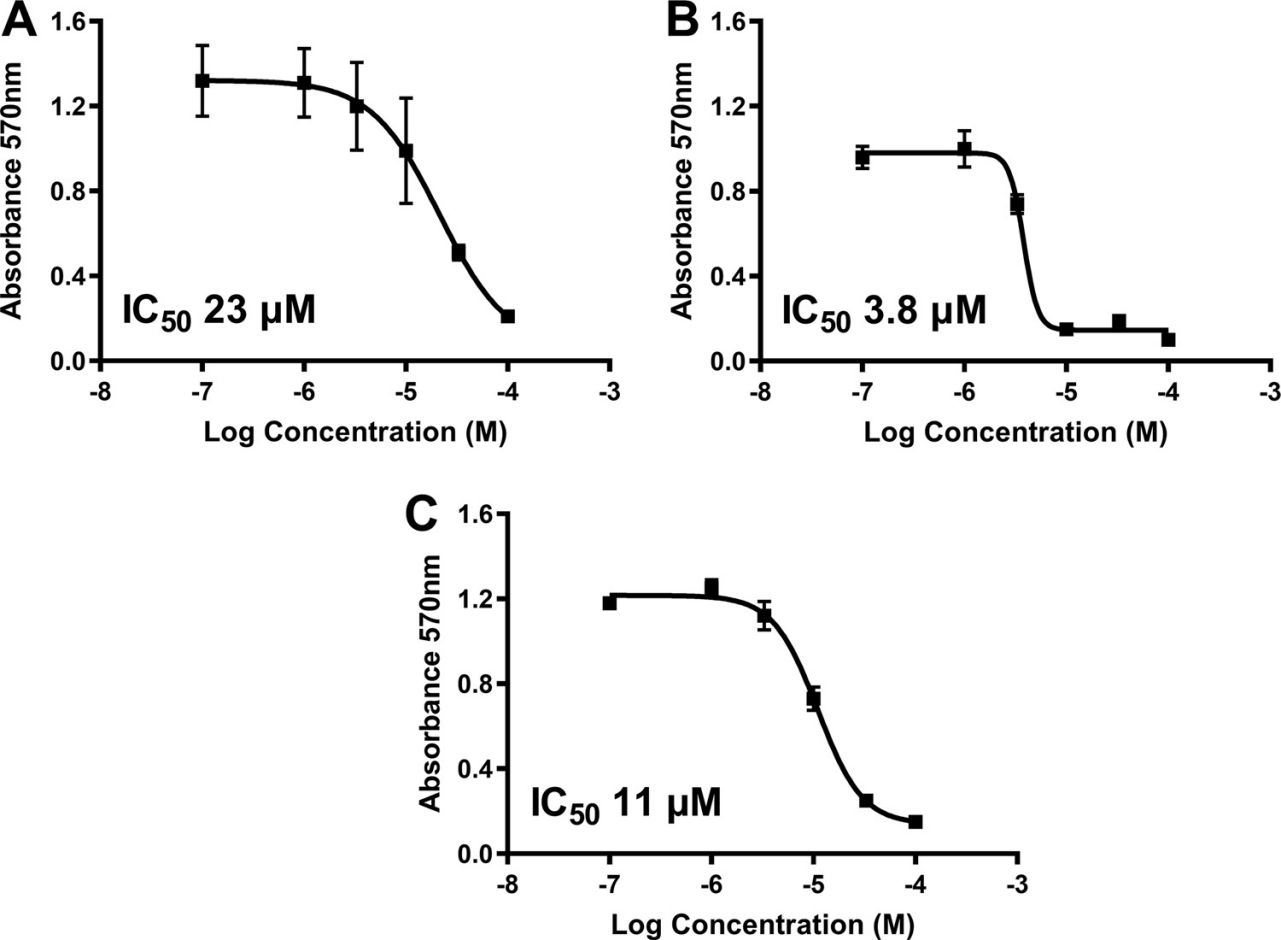
Allantodapsone inhibits S. aureus Newman adhesion to fibrinogen. Dose response curves for S. aureus Newman (A), L. lactis ClfA (B), and L. lactis ClfB (C) adhesion to fibrinogen. Fibrinogen was used to coat plates at 4°C overnight at 10 μg/mL. Wells were washed with PBS and blocked with 1% BSA. Washed bacteria (OD_600_ of 1) were mixed with the relevant concentration of allantodapsone or vehicle control (DMSO) for 5 min, and added to the wells, incubating for 2 h at 37°C. Adherent cells were washed, fixed, and stained with crystal violet, and wells further washed. The dye was dissolved in acetic acid and the absorbance measured at 570 nm. IC_50_ values were generated using GraphPad Prism 9.0 GraphPad software, San Diego, CA. Data are representative of three experiments with error presented as SEM.

**FIG 2:**
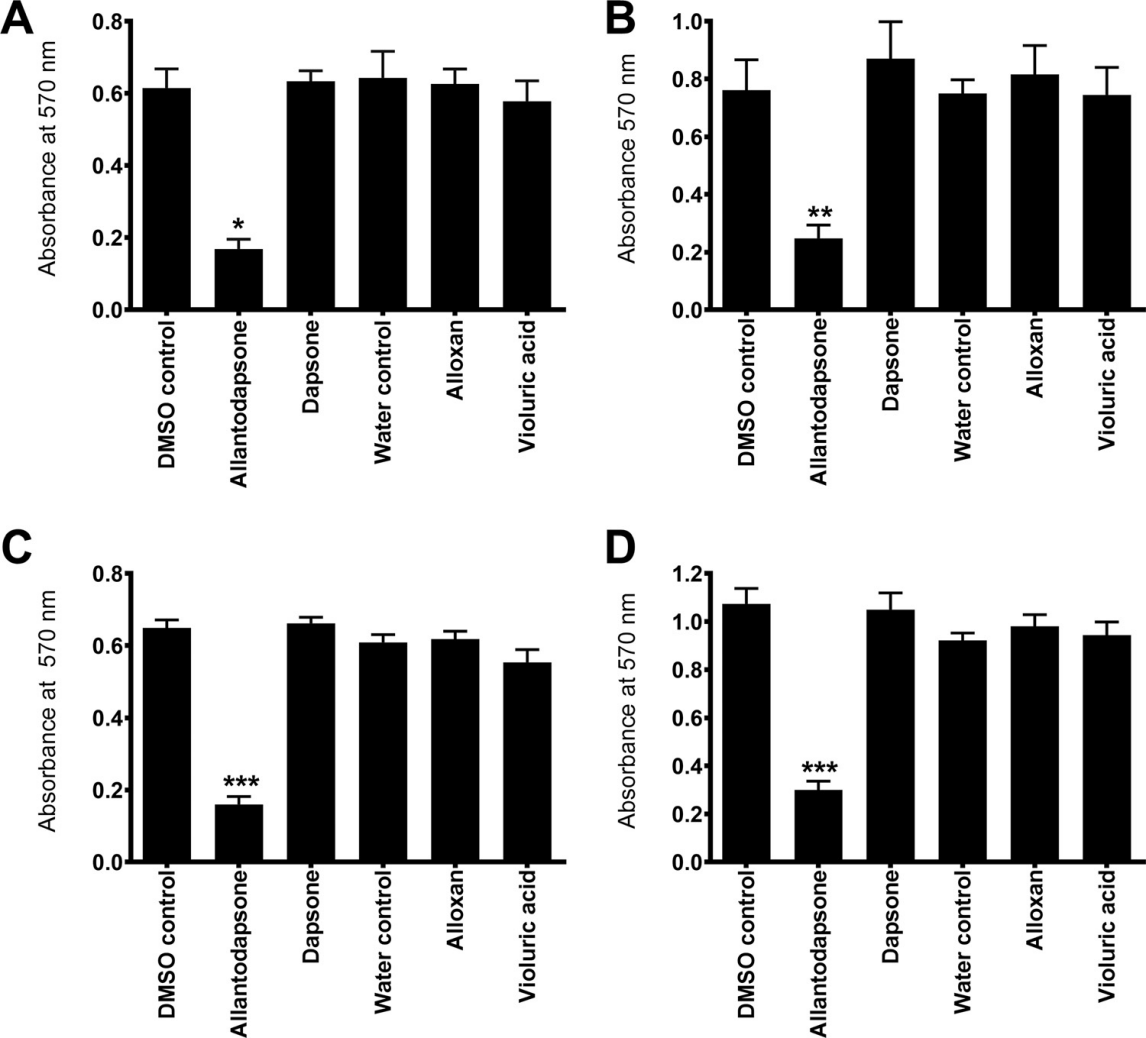
Allantodapsone inhibits S. aureus adhesion to fibrinogen. (A) S. aureus Newman stationary phase and (B) exponential phase. (C) S. aureus SH1000 stationary phase and (D) exponential phase bacteria. Fibrinogen was used to coat plates at 4°C overnight at 10 μg/mL. Wells were washed with PBS and blocked with 1% BSA. Washed bacteria (OD_600_ of 1) were mixed with the relevant concentration of allantodapsone or vehicle control for 5 min, and added to the wells, incubating for 2 h at 37°C. Adherent cells were washed, fixed, and stained with crystal violet, and wells further washed. The dye was dissolved in acetic acid and the absorbance measured at 570 nm. Compounds were screened at a concentration of 70 μM and compared to their vehicle control. Statistical analysis was carried out using repeated measures ANOVA followed by multiple comparisons Dunnett’s tests using GraphPad PRISM. **, *P* ≤ 0.01; ***, *P* ≤ 0.001; ****, *P* ≤ 0.0001 (*n* = 3).

*In vitro* screening of compounds from the ZINC database (16) led to the initial identification of allantodapsone as an inhibitor of S. aureus Newman adhesion to fibrinogen (Fig. 1). The virulence factors targeted in this study are expressed in both MSSA and MRSA strains (17). S. aureus Newman is a clinically derived strain, with well characterized expression of ClfA and ClfB (18, 19). The ability of allantodapsone to inhibit S. aureus Newman growing to stationary phase was initially tested. Under these conditions, ClfA is the dominant MSCRAMM on the bacterial surface and there is little or no ClfB expression (6). Allantodapsone caused a dose dependent inhibition of adhesion of S. aureus to immobilized fibrinogen with an IC_50_ of 21.3 μM (95% CI 4.5–102 μM), minimum adhesion inhibitory concentration (MAIC) of 100 μM (40.2 μg/mL) (Fig. 1A). L. lactis has been employed as a surrogate Gram-positive bacterial host to express individual MSCRAMMs in isolation (19). ClfA and ClfB can be expressed individually on the surface of this surrogate host. Both ClfA and ClfB have been shown to be expressed on the surface of L. lactis using this expression system and functionally able to support adhesion to fibrinogen where the L. lactis wild-type host strain does not (19). L. lactis expressing ClfA and L. lactis expressing ClfB display similar adhesion levels to S. aureus Newman and S. aureus SH1000 strains. Comparative adhesion to fibrinogen for strains used in this study is presented in Fig. S1 in the supplemental material. Allantodapsone inhibited adhesion of L. lactis ClfA to fibrinogen with an IC_50_ of 3.8 μM (95% CI 1.0–14.3 μM; MAIC 10 μM; 4.0 μg/mL) (Fig. 1B) and L. lactis ClfB adhesion to fibrinogen with an IC_50_ of 11.0 μM (95% CI 8.9–13.6 μM; MAIC 33 μM; 13.3 μg/mL) (Fig. 1C). Allantodapsone also inhibited adhesion to fibrinogen by Newman grown to the exponential phase (Fig. 2). S. aureus strain SH1000 produces two additional fibrinogen binding proteins not expressed by Newman, fibronectin binding protein A (FnBPA) and FnBPB. Adhesion of strain SH1000 to fibrinogen in both the stationary and exponential growth phases was inhibited by allantodapsone (Fig. 2). Comparative results for all strains is presented in Table 1. Further assays demonstrated that allantodapsone did not inhibit adhesion of S. aureus SH1000 to fibronectin (Fig. S2).

**TABLE 1:** Summary of IC_50_ values for inhibition of adhesion to fibrinogen^*a*^

Bacteria	IC_50_ (μM)	95% confidence interval (μM)
S. aureus Newman (exponential phase)	32.8	21.3–44.9
S. aureus Newman (stationary phase)	21.3	4.5–102
S. aureus SH1000 (exponential phase)	15.6	10.6–21.0
S. aureus SH1000 (stationary phase)	7.6	4.8–10.0
L. lactis ClfA	3.8	1.0–14.3
L. lactis ClfB	11.0	8.9–13.6

a IC_50_ values were generated using GraphPad Prism 9.0 (GraphPad Software, San Diego, CA).

### Investigating substructures of allantodapsone.

Allantodapsone is a composite molecule formed from dapsone and alloxan substructures. The individual components were tested for inhibition of adhesion of S. aureus Newman and strain SH1000 growing to exponential phase and to stationary phase. Neither dapsone nor alloxan had any inhibitory activity at the concentration of allantodapsone that reduced adhesion of S. aureus strains to fibrinogen (Fig. 2).

### Investigation of effects on bacterial growth.

**FIG 3:**
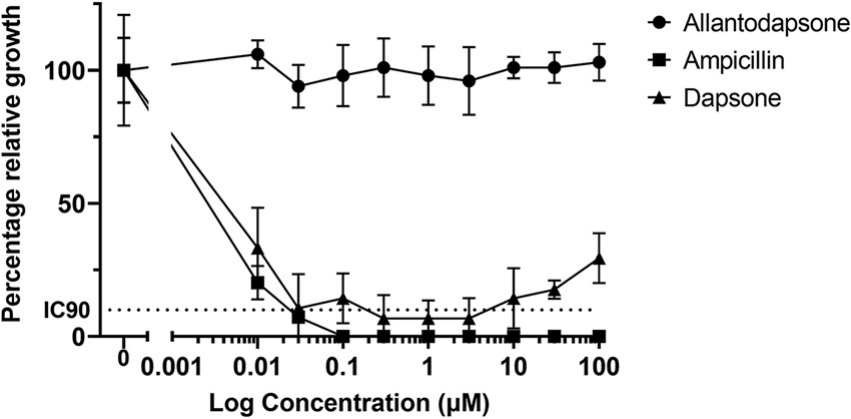
Allantodapsone does not inhibit bacterial growth. S. aureus was grown in BHI broth shaking overnight at 37°C in the presence of a range of concentrations of allantodapsone, dapsone, or ampicillin as control. Absorbance was measured the following day at 570 nm. Data have been normalized to the relevant vehicle control (1% DMSO for allantodapsone and dapsone and water for ampicillin). These data represent three separate experiments. Error bars represent SEM.

Alloxan is known to have toxic effects (20, 21); therefore, we tested allantodapsone to assess if it had any effects on bacterial growth. In order to determine if allantodapsone had growth inhibitory properties, S. aureus Newman cells were diluted into fresh broth containing different concentrations of allantodapsone and incubated to allow growth to occur (Fig. 3). Even at the highest concentration of allantodapsone tested (100 μM), there was no inhibition of bacterial growth compared to the vehicle control. In contrast, the antibiotic ampicillin had a pronounced inhibitory effect on growth. The MIC_90_ in this assay for S. aureus Newman was 0.03 μM (0.01 μg/mL). The MIC_90_ for ampicillin is reported for S. aureus isolates in the range of 0.015–4 μg/mL, using the microdilution method in MSSA isolates (22, 23). Dapsone also inhibited growth with an MIC_90_ of 0.3 μM (0.1 μg/mL). An MIC_90_ for dapsone was previously determined in MSSA strains as 128 μg/mL (32–256 μg/mL) (24). A preliminary toxicity test was performed in human cells where no toxic effects were seen when allantodapsone was tested at a 100 μM concentration (Fig. S3).

### Inhibition of adhesion to loricrin and cytokeratin 10.

The ability of S. aureus Newman to adhere to loricrin and cytokeratin 10 is crucial for colonization of the nares of carriers and the skin of eczema patients. Adhesion is mediated exclusively by ClfB (11). The *in vitro* expressed recombinant GST-L2V protein contains a high affinity binding site for ClfB from human loricrin.

**FIG 4:**
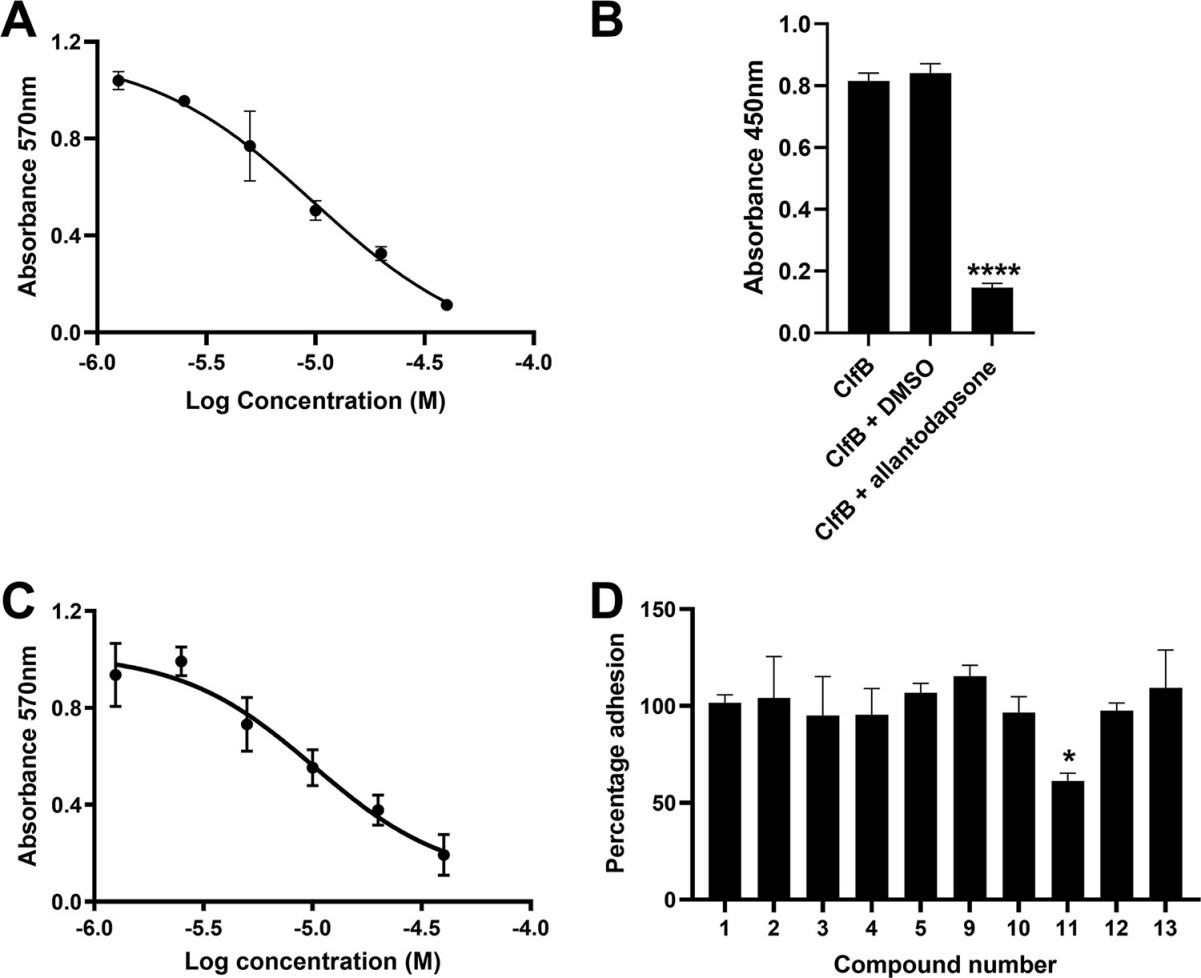
Allantodapsone inhibits S. aureus adhesion to loricrin and cytokeratin 10. Dose-response curve for adhesion of S. aureus Newman to GST-L2v loricrin (A) and CK10 (C). (B) Inhibition of recombinant ClfB N2N3 binding to immobilized GST-L2v loricrin by allantodapsone. (D) Inhibition of S. aureus Newman adhesion to GST-L2v loricrin by allantodapsone derivatives. Purified GST-L2v or CK10 were used to coat plates at 4°C overnight. Wells were washed with PBS and blocked with 1% BSA. Washed bacteria (OD_600_ of 1) were mixed with allantodapsone or vehicle control before being added to the wells and incubated for 2 h at 37°C. Adherent cells were washed, fixed, and stained with crystal violet, and wells further washed. The dye was dissolved in acetic acid, and the absorbance measured at 570 nm. GST-L2v was used at 20 μM in A and D. CK10 was used at 10 nM. Test compounds were incubated with bacteria at 80 μM and normalized to the DMSO vehicle control in D. For data presented in B, recombinant ClfB was incubated with allantodapsone (100 μM) or DMSO (1%) for 30 min prior to being added to wells coated with recombinant GST-L2v loricrin. Bound ClfB was detected using peroxidase-conjugated anti-His monoclonal antibody in an ELISA. The graphs shown are representative of three independent experiments. Statistics were carried out using one-way ANOVA followed by the Dunnett’s posttest using GraphPad Prism version 9.0.

Figure 4A demonstrates that allantodapsone caused a dose dependent inhibition of S. aureus adhesion to GST-L2v (the high affinity ClfB binding site within loricrin), with full inhibition at 40 μM (OD_600_ 0.11 ± 0.01). Furthermore, recombinant ClfB binding to L2v loricrin was inhibited by allantodapsone (*P < *0.0001) (Fig. 4B). In this experiment, the N2 and N3 subunits of the ligand binding A-domain of ClfB (ClfB N2N3) was used, demonstrating specificity for this domain on ClfB. Allantodapsone also demonstrated dose dependent inhibition of S. aureus adhesion to CK10 (Fig. 4C).

### Structural analysis and derivative design.

**FIG 5:**
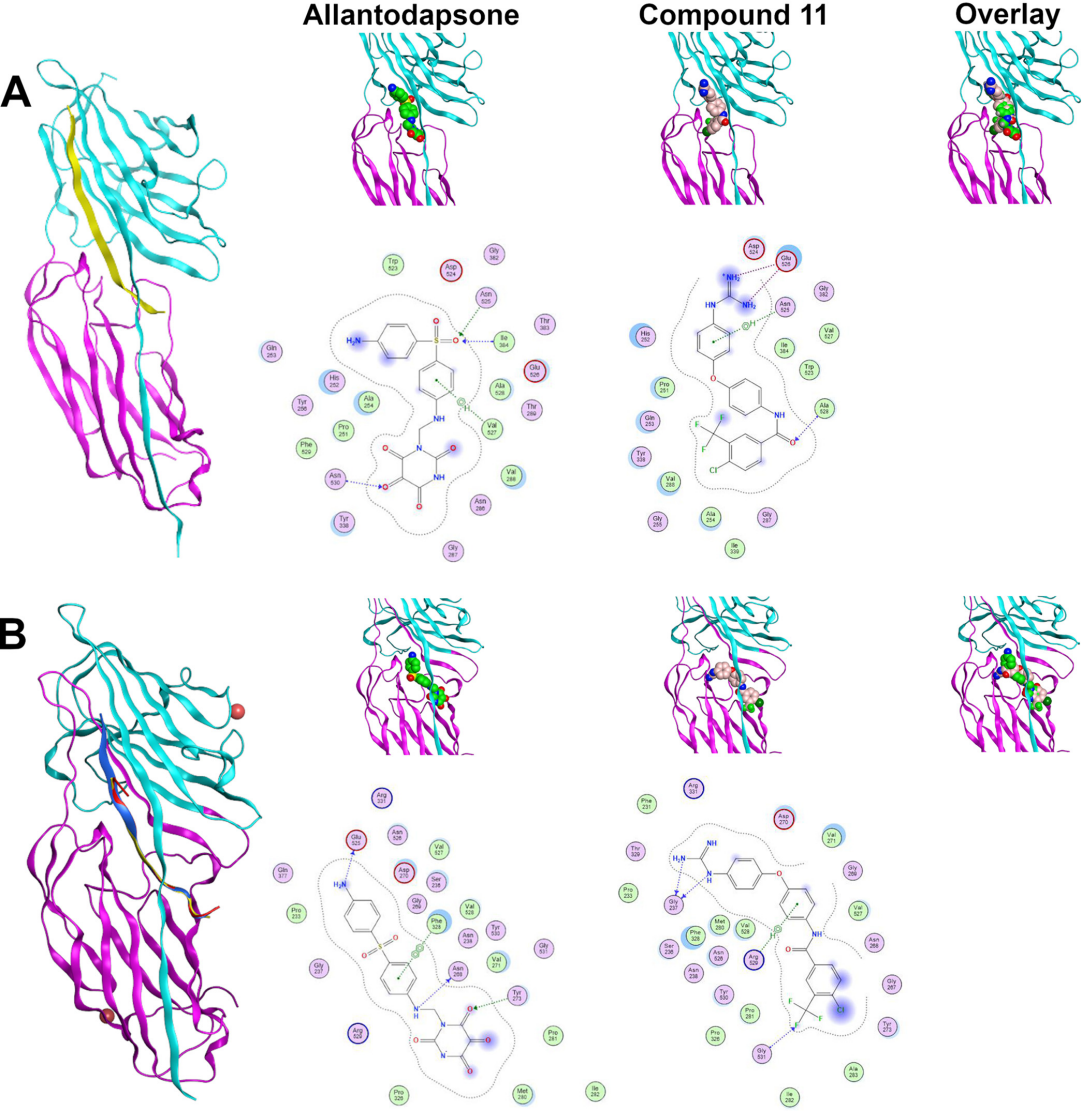
Predicted binding interactions for allantodapsone and compound 11 with ClfA and ClfB. Panel A represents predicted ligand interactions with ClfA. The co-crystal structure (PDB 2VR3) is presented with the fibrinopeptide depicted as a yellow ribbon orientating the binding site. The carbons of allantodapsone are colored green, and the carbons of compound 11 are colored pink. Oxygen is colored red, nitrogen-blue, chlorine-dark green, and fluorine-bright green. The final column presents an overlay of the two compounds. Panel B represents ClfB structures. The ClfB co-crystal structure peptide ligands are predicted to bind to the same binding site in the groove between the N2 and N3 of the A domain. Superposition of ClfB crystal structures with peptide ligands was performed using MOE (Chemical Computing Group, Montreal). ClfB and the peptide ligands are presented as ribbon structures. ClfB is presented with the N-terminus in purple and the C-term in cyan. The dermokine peptide is presented in yellow, CK10 peptide in blue, and the fibrinogen alpha chain peptide in red. The structures used for this overlay are PDB 4F20, 4F1Z, and 4F27. Magnesium ions are depicted as space-fill and colored pink. The zoomed-in panels represent the predicted binding poses for allantodapsone and compound 11 to ClfB (PDB 4F20). The small molecules are depicted as space-fill with hydrogen atoms hidden. The final column represents the overlay of the two best docking poses for allantodapsone and compound 11.

As individual fragments of allantodapsone (dapsone, alloxan, and violuric acid) did not show any activity, we sought to better understand the allantodapsone structure–activity relationship and to improve its biological activity. Thus, a series of novel allantodapsone related compounds were designed and their interaction with ClfA and ClfB computationally analyzed by docking studies using the crystallized structure of ClfA and ClfB as templates (PDB: 2VR3 and 4F20). The fibrinogen γ-chain peptide has been co-crystalized with ClfA binding between the N2 and N3 domains as depicted in Fig. 5A (13). CK10, dermokine, and α-chain fibrinogen peptides have been cocrystalised with ClfB (13, 15), demonstrating that they occupy the similar peptide binding groove between the N2 and N3 domains of the A domain of ClfB. An overlay of the three structures is presented in Fig. 5B, demonstrating that these peptides share the same binding site. Our docking studies predicted that allantodapsone binds into the same peptide binding groove in both ClfA and ClfB (Fig. 5), suggesting that it would be able to directly compete with the natural peptide ligands.

In terms of the novel derivatives proposed, structural modifications considered to elucidate and improve its interaction with the target involved (i) changing the sulfur atom oxidation state from sulfone to sulfide to investigate the role of the two sulfoxide groups upon binding to the target (compounds 1–5, 7–8), (ii) modifying the spacer between the dapsone portion and the alloxan-like moiety by increasing the carbon chain length (compounds 1–3), (iii) connecting flexible chains to the dapsone core by means a urea/thiourea linker (6–8), as well as (iv) reducing the flexibility of the system introducing an amide group or introducing a spiro function (compounds 2–5) (see structures in Table 2). Additionally, a number of compounds with a similar diaromatic core as allantodapsone were selected from Rozas’ library; these compounds contain different bis-guanidinium diaryl derivatives 3,4’- or 4,4’-substituted and with additional aromatic systems in one of the guanidinium groups (compounds 9–13; see structures in Table 2).

**TABLE 2:** Summary of chemical derivatives synthesized and tested with docking results for ClfA and ClfB^*a*^

Name	Structure	ClfA docking score (kcal/mol)	ClfB docking score (kcal/mol)
Allantodapsone	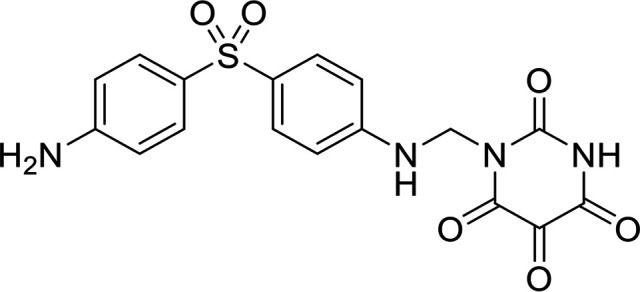	−8.05	−7.15
1	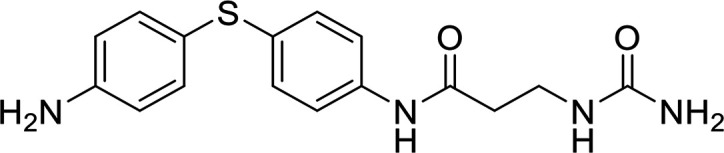	−7.22	−7.13
2	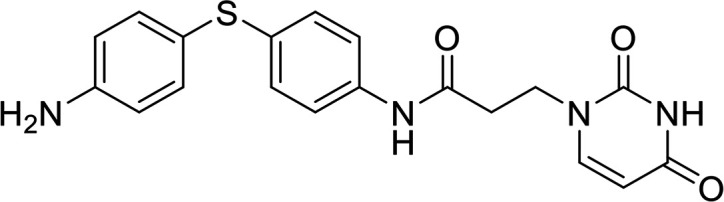	−7.80	−7.72
3	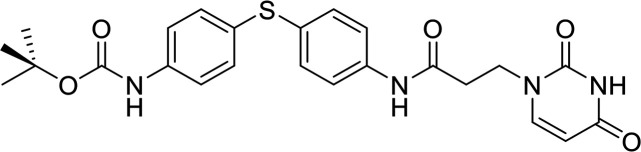	−9.32	−8.43
4	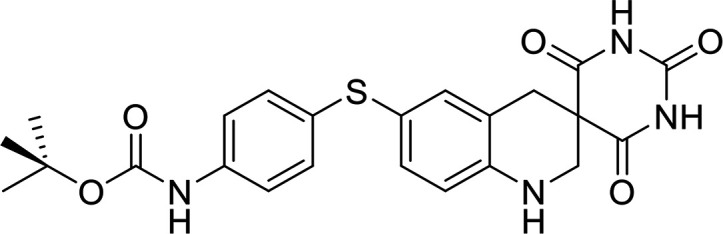	-8.93	-7.29
5	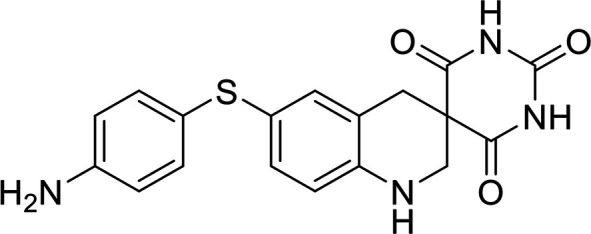	−7.76	−6.27
6	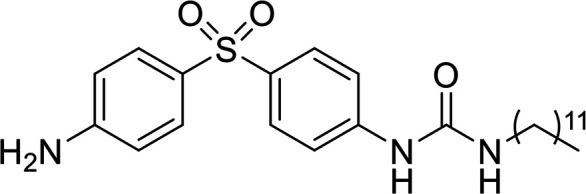	−9.77	−8.72
7	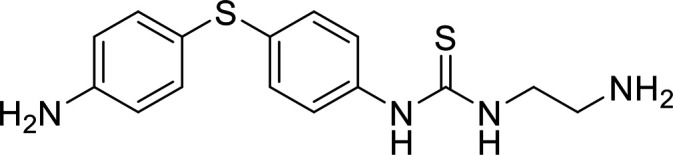	−7.01	−6.41
8	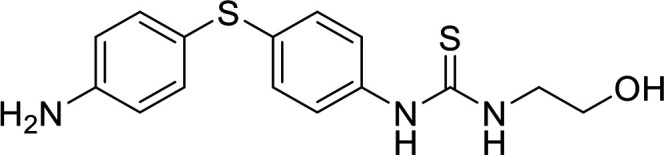	−7.13	−6.33
9	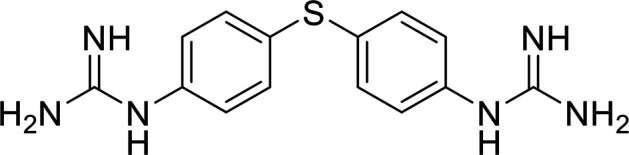	−6.95	−6.30
10	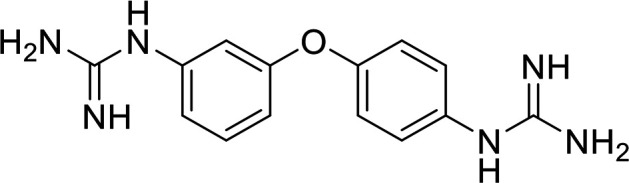	−7.03	−6.63
11	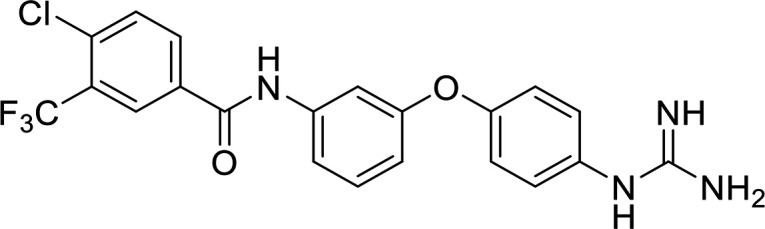	−7.95	−7.53
12	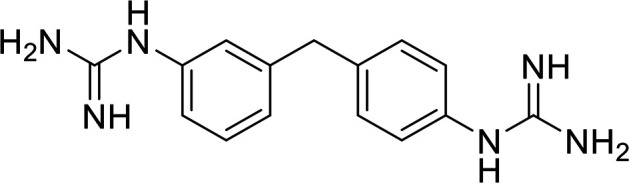	−6.96	−6.25
13	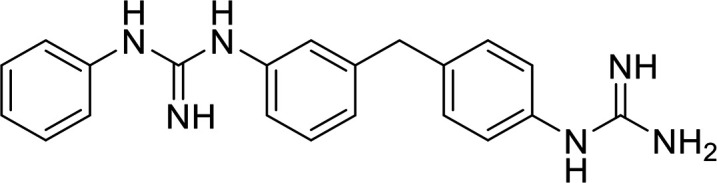	−8.14	−7.34

a Scores obtained from docking all the compounds studied into ClfA and ClfB are presented. Proteins were prepared using Proteinate3D and docking performed using the dock function in MOE (CCG, Montreal, Canada). PDB structures 2VR3 and 4F20 were used for ClfA and ClfB docking, respectively.

Molecular docking was used to explore the predicted structure activity relationships for ClfA and ClfB, and the results are presented in Table 2. All compounds showed scores similar or even better than those of allantodapsone, and accordingly they were deemed to be potential ClfA and ClfB inhibitors and their synthesis was attempted.

These new compounds (1–7), together with those selected from Rozas’ library (8–13, 25), were screened for activity using adhesion of S. aureus Newman to L2v loricrin at 80 μM (Fig. 4D). Only compound 11 demonstrated activity (61.3% ± 7% adhesion); however, this was not as potent as allantodapsone, which has an IC_50_ of 9.87 μM in this assay and full inhibition at 40 μM (16.1 μg/mL) (Fig. 4A). Compounds 6–8 were also screened at 40 μM and showed no significant inhibition (data not shown). Compound 11 has been previously shown to exert toxicity in human cancer cells such as HL-60, HeLa, and MCF-7 in the micromolar concentration range (IC_50_ values between 11 and 28 μM) (26), suggesting that this compound would also have other off-target effects.

To understand these outcomes, we analyzed the docking results obtained with compound 11 and allantodapsone with ClfA and ClfB. The best pose of allantodapsone bound to ClfA predicts the formation of a specific hydrogen bond with the side chain of Asn^525^ and with the peptide backbone of Asn^530^ and Ile^384^, while the ring structure of the dapsone substructure has a predicted arene-H interaction with Val^527^ (Fig. 5A). While compound 11 binds to the same general region (overlay panel), it is predicted to interact with different amino acids. The guanidinium group of compound 11 is predicted to form a salt bridge with the Glu^526^; additionally, it is also predicted to have a backbone interaction with Ala^528^, and an arene-H interaction with Asn^525^.

Glu^526^ and Val^527^ have been shown to be important for ClfA adhesion to fibrinogen (27), Tyr^338^ has also been demonstrated to be crucial for fibrinogen binding (3, 28). Both compound 11 and allantodapsone top poses are near to Tyr^338^, suggesting that they would block this interaction.

In the case of docking to ClfB, predicted hydrogen bonding occurs through Asn^268^, Tyr^273^, and Glu^525^, with a pi-pi interaction predicted with Phe^328^ for allantodapsone. Phe^328^ was shown to be an important residue in the interaction between ClfB with CK10 and dermokine (15).

Compound 11 is predicted to form hydrogen bonds between the guanidinium group and Gly^237^ and with Gly^531^, as well as an arene-H interaction with Arg^529^. The top docking poses for both allantodapsone and compound 11 are near to Ser^236^, which has been shown to be important for binding of fibrinogen dermokine and CK10 (15). Both compounds are predicted to completely block the peptide binding site and therefore are foreseen to inhibit interactions with all peptide ligands binding to the binding trench.

Based on the experimental data, allantodapsone is the most promising inhibitor of S. aureus adhesion identified in this work. As ClfA and ClfB are also important MSCRAMM’s involved in the activation of platelets, important in the pathogenesis of sepsis, we sought to investigate if allantodapsone had any effect on bacterial-induced platelet activation. Inhibition of this interaction therefore could be beneficial to patients. We demonstrated that allantodapsone did not inhibit platelet aggregation induced by S. aureus Newman (Fig. S4A). We further investigated whether allantodapsone could inhibit soluble fibrinogen binding using the agglutination assay. S. aureus Newman was washed and resuspended in fibrinogen in order to investigate the clumping factors without the presence of coagulase that is secreted. Allantodapsone did not inhibit clumping of bacteria in a fibrinogen clumping assay (Fig. S4B). These results together suggest that allantodapsone does not inhibit the interaction of soluble fibrinogen with ClfB and that the inhibition demonstrated is limited to immobilized fibrinogen.

## DISCUSSION

Allantodapsone was originally identified as an inhibitor of protein arginine methyltransferase 1 (PRMT1) from virtual high throughput screening (29). We have shown that allantodapsone inhibits adhesion of ClfB to fibrinogen, CK10, and L2v loricrin, making it an ideal candidate for development as an inhibitor for nasal colonization or for atopic dermatitis. It further inhibits both ClfA and ClfB adhesion to fibrinogen, indicating that it may also be useful for inhibition of wound colonization. It inhibits adhesion of both S. aureus Newman and SH1000 to fibrinogen. S. aureus SH1000 expresses the fibronectin binding proteins FnBPA and FnBPB that bind to fibronectin as well as fibrinogen. Thus, our results suggest that allantodapsone is an inhibitor of the clumping factors A and B as well as the fibronectin binding proteins FnBPA and FnBPB binding to fibrinogen. The lack of inhibition of S. aureus SH1000 to fibronectin suggests that there is some specificity. The fibronectin binding site is distinct from the fibrinogen binding site on the fibronectin binding proteins, and therefore this is not unexpected, as FnBPA, FnBPB, and ClfA also share the same ligand binding sequence in fibrinogen. ClfB and FnBPB have been recently shown to interact with corneodesmosin on the surface of deformed corneocytes in inflamed eczema skin (30). Therefore, further work to investigate the effect of allantodapsone on this interaction is required.

Unexpectedly, allantodapsone did not inhibit platelet aggregation or clumping of S. aureus in a solution of fibrinogen, suggesting that it does not inhibit the interaction of S. aureus with soluble fibrinogen. The reasons for this are not clear, but the high affinity binding of ClfA to fibrinogen involves both docking of the fibrinogen γ-chain in the hydrophobic binding trench (11, 31) and another interaction at a second distinct site at the top of the N3 subdomain. Based on our predicted docking site, allantodapsone is unlikely to interfere with this second site interaction on the N3 subdomain. Ganesh et al. (32) found that the reverse was true, with a monoclonal antibody recognizing an epitope overlapping with the second fibrinogen binding site on N3, inhibiting binding to soluble fibrinogen to a greater extent than immobilized fibrinogen. They showed that the antibody did not inhibit binding of the fibrinogen γ-chain to the hydrophobic trench where allantodapsone is predicted to bind.

While the allantodapsone molecule itself does not appear to have toxic side effects, there is some concern as to the potential toxicity of the alloxan and dapsone fragments. We therefore sought to alter these components. While complete loss of activity was seen in most compounds screened, compound 11 maintained some activity providing us with a possible new scaffold for development. Docking studies suggest that the overall position is similar for compound 11 and allantodapsone, but specific interactions are different. While compound 11 had slightly reduced activity compared to allantodapsone, it is possible that extending the molecule further along the binding groove may increase the ligand interactions with the protein and therefore improve activity. Replacement of the 4-chloro-3-(trifluoromethyl)benzamide group with a phenylguanidinium group (i.e., compound 10) resulted in a loss of activity for this new scaffold, suggesting that this hydrophobic group is important for specificity. In the modeling of compound 11, this group was predicted to form a hydrogen bond in the ClfB structure.

Here we present novel inhibitors of the MSCRAMMs, ClfA, and ClfB. Identification of novel inhibitors of the clumping factors is enticing as development of resistance to this class of molecules would be less likely, as any loss of binding to the compound would also lead to loss of ability to colonize the host due to disruption of the binding interaction with the natural ligand. This ligand binding region between the N2 and N3 subdomains has been shown to be completely conserved in ClfA and ClfB in both MSSA and MRSA strains (33). Therefore, a major advantage of adhesion blockers, compared to antibiotics, is that there is no selective advantage for development of resistance. The surface-located clumping factors are expressed ubiquitously in clinical strains including antibiotic resistance strains (2, 17). Therefore, while our experiments were performed using methicillin sensitive strains, and L. lactis expressing the clumping factors, activity against all S. aureus strains expressing the clumping factors is a reasonable expectation. These surface proteins are crucial for host colonization. Furthermore, as this is a new target that has not been previously exploited by antibiotics, there are no current antibiotic resistance mechanisms to similar drugs.

### Conclusion.

Allantodapsone inhibits S. aureus adhesion to fibrinogen and the ClfB ligands loricrin and cytokeratin 10. Loricrin binding to ClfB has been implicated as the primary mechanism that S. aureus uses to colonize the nares. ClfB also facilitates adherence of S. aureus to corneocytes in the stratum corneum to initiate skin colonization (31). Allantodapsone therefore has potential for development as a novel therapeutic for the prevention of skin colonization in atopic dermatitis, wound colonization, and nasal colonization.

The *in vitro* work presented here demonstrates that it is possible to inhibit the interaction between the MSCRAMM’s ClfA, ClfB, and their ligands cytokeratin 10, loricrin and fibrinogen. Allantodapsone has potential for development as a colonization inhibitor; however, further testing using clinically relevant strains and in *in vivo* models is required.

## MATERIALS AND METHODS

### Molecular modeling.

ClfB structures were downloaded from the Protein Data Bank (34). The structures for ClfB, PDB ID 4F20, 4F1Z and 4F27, were used (15). ClfB structures and their ligands were superposed using the align/superpose function in the Molecular Operating Environment (Chemical Computing Group, Montreal, Canada). All ligands studied were prepared for docking using the “wash” function and all tautomers enumerated. CCPDB structures, 4F20 (ClfB) (15) and 2VR3 (ClfA) (13) were used as the targets and prepared for docking using the protonate 3D function with default parameters. The peptide ligand was used to define the binding site for docking using macromolecule residues within 4.5 Å of the peptide ligand. Docking was performed using the Triangle Matcher function for placement and London delta G scoring, storing 30 poses. Rigid receptor was used for refinement and GBVI/WSA delta G scoring for the final poses.

### Chemistry.

General conditions: All commercial chemicals were obtained from either Sigma-Aldrich or Fluka and used without further purification. Deuterated solvents for NMR spectroscopy use were purchased from Apollo. Column chromatography was performed using Sigma-Aldrich silica gel 100–200 mesh. Solvents for synthesis purposes were used at GPR grade. Analytical TLC was performed using either Merck Kieselgel 60 F254 silica gel plates or Polygram Alox N/UV254 aluminum oxide plates. Visualization was by UV light (254 nm). NMR spectra were recorded on Bruker DPX-400 Avance spectrometers, operating at 400.13 and 600.1 MHz for ^1^H NMR, 100.6 and 150.9 MHz for ^13^C NMR. Shifts are referenced to the internal solvent signals.^[1]^ NMR data were processed using BrukerTOPSPIN software. HRMS spectra were measured on a Micromass LCT electrospray TOF instrument with a WATERS 2690 autosampler and methanol/acetonitrile as carrier solvent. Melting points were determined using a Stuart SP10 melting point apparatus and are uncorrected. Infrared spectra were recorded on a PerkinElmer Spectrum One FT-IR spectrometer equipped with a Universal ATR sampling accessory. HPLC purity analysis was carried out using a Varian ProStar system equipped with a Varian Prostar 335 diode array detector and a manual injector (20 μL). For purity assessment, UV detection was performed at 254 nm and peak purity was confirmed using a purity channel. The stationary phase consisted of an ACE 5 C18-AR column (150 × 4.6 mm), and the mobile phase used the following gradient system, eluting at 1 mL/min: aqueous formate buffer (30 mM, pH 3.0) for 10 min, linear ramp to 85% methanol buffered with the same system over 25 min, hold at 85% buffered methanol for 10 min. Minimum requirement for purity was set at 95.0%. Allantodapsone was provided as a kind gift from the National Cancer Institute (Bethesda, USA). Alloxan, violuric acid, and dapsone were purchased from Sigma-Aldrich, United Kingdom. Details of the preparation and characterization of all compounds studied are presented in the Supplemental Material.

### Microbiology.

**(i) Bacterial strains.** Strains used in this study are listed in Table 3. S. aureus strain Newman NCTC8178 was isolated from a patient suffering from osteomyelitis (18). It has been widely used to study S. aureus surface proteins and their roles in pathogenesis (19, 35). S. aureus strain SH1000 is derived from laboratory strain 8325-4 with a defect in *rsbU* restored to wild type (36). L. lactis strain MG1363 was employed as a surrogate host for constitutive expression of S. aureus surface proteins ClfA and ClfB from the erythromycin resistance conferring plasmid pKS80 (19). S. aureus Newman (18) and the S. aureus strain 8325-4 SH1000 (36) were used to assess fibronectin binding. L. lactis MG1363 was used for heterologous expression of ClfA and ClfB (19).

**TABLE 3:** Summary of bacterial strains utilized in this study

Bacterial strain	Genotype or description	Reference
L. lactis control	Strain MG1363	Gasson et al. (38)
L. lactis ClfA	Strain MG1363 expressing plasmid pKS80 carrying the *clfA* gene	O’Brien et al. (19)
L. lactis ClfB	Strain MG1363 expressing plasmid pKS80 carrying the *clfB* gene	O’Brien et al. (19)
S. aureus Newman	NCTC8178, clinical isolate	Duthie et al. (18)
S. aureus SH1000	Derivative of strain 8325-4, *rsbU*+	Horsburgh et al. (36)

**(ii) Bacterial growth and preparation.** Staphylococcus aureus Newman and SH1000 were grown in brain heart infusion (BHI) broth (Oxoid, UK), at 37°C with orbital shaking at 220 rpm. L. lactis MG1363 wild type and L. lactis expressing ClfA and ClfB were grown as static cultures at 30°C in M17 broth (Oxoid, UK) supplemented with 0.5% glucose and 5 μg/mL erythromycin.

Bacterial growth curves were used to ascertain when cultures had reached exponential phase. Single colonies were used to generate starter cultures overnight, which were then used to inoculate larger volumes of broth. Samples were taken every 30 min and absorbance measured at 600 nm using the Ultrospec III spectrophotometer (Pharmacia Biotech UK), to determine when the cultures had reached exponential phase. A single colony was used to inoculate overnight broth cultures used to prepare bacteria for experiments that required stationary phase bacteria.

Bacterial cultures were centrifuged at 5,000 × *g* at room temperature for 10 min. The broth was discarded and the pellets washed in PBS and centrifuged again at 5,000 × *g* for a further 10 min. The resulting pellets were resuspended in PBS to the relevant optical density (OD), measured using the Ultrospec III spectrophotometer (Pharmacia Biotech, UK), at 600 nm. OD_600_ 1.6 was used for platelet aggregations, and OD_600_ of 1.0 was used for adhesion assays. The optical density, (OD_600_) of 1.6 corresponds to approximately 7 × 10^9^ cells, and OD_600_ of 1.0 corresponds to approximately 3 × 10^9^ cells (determined using CFU counts).

### (iii) Bacterial adhesion assay.

Adhesion assays were carried out according to Hartford et al. (37). Briefly, plates were coated with 10 μg/mL fibrinogen (Calbiochem, Darmstadt, Germany) or 20 μg/mL fibronectin (Sigma-Aldrich, Ireland) for 2 h at 37*°*C or for 16 h at 4°C. Plates were blocked with 1% BSA at 37°*C* for 1 h and washed 3 times in PBS. Washed bacteria were incubated at 37°C for 2 h. Adherent bacteria were washed three times in PBS and fixed in 5–9% formaldehyde at room temperature for 30 min. Wells were washed three times in PBS and stained using 5% crystal violet for 20 min. Wells were washed three times in PBS, and the crystal violet solubilized using 5% acetic acid. Plates were read at 570 nm on a Wallac Victor_2_, (Shelton, CT, USA). The minimum adhesion inhibitory concentration (MAIC) was determined as the concentration inhibiting greater than 90% bacterial adhesion.

### (iv) Compound screening.

Compounds were added to the bacterial suspension prior to addition to the wells. In each experiment, vehicle controls were prepared using the same volume of vehicle for the matching test compound.

### (v) Antimicrobial assay.

S. aureus was grown overnight in BHI broth at 37°C in shaking incubator. Wells were inoculated with 10 μL of overnight culture into 90 μL of broth containing the test compounds or controls. A growth control containing no agents was used (positive control), a sterility control of broth alone was included, and vehicle controls were included for each test compound. Plates were incubated overnight at 37°C and absorbance measured at 570 nm the following day using a Wallac Victor_2_, (Shelton, CT, USA).

### (vi) ELISA.

Recombinant GST-tagged L2v loricrin (120 nM) was coated onto a microtiter plate (Nunc Maxisorb) in sodium carbonate buffer (15 mM Na_2_CO_3_, 35 mM NaHCO_3_, pH 9.6) for 16 h at 4°C. Wells were washed three times with PBS and were incubated for 2 h at 37°C with 5% (wt/vol) skimmed milk proteins in PBS to block nonspecific binding. Wells were washed again, and recombinant His-tagged ClfB alone (5 μM), with allantodapsone (100 μM) or with DMSO was added to the coated wells. Plates were incubated for 1 h at room temperature with shaking. Bound ClfB was detected with peroxidase-conjugated anti-His monoclonal antibody in 1% (wt/vol) skimmed milk proteins in PBS. After washing, 100 μL of chromogenic substrate solution (1 mg/mL tetramethylbenzidine and 0.006% H_2_0_2_ in 0.05 M phosphate citrate buffer pH 5.0) was added, and plates were developed for 10 min in the dark. The reaction was stopped by the addition of 2 M H_2_S0_4_ (50 μL/well), and the A_450_ was measured in a plate reader (Labsystems).
