# Supplementary material for: Allantodapsone is a Pan-Inhibitor of Staphylococcus aureus Adhesion to Fibrinogen, Loricrin, and Cytokeratin 10
Source: Microbiol Spectr. 2022 Jun 1;10(3):e01175-21. doi: 10.1128/spectrum.01175-21 (PMC9241669; doi:10.1128/spectrum.01175-21)
Supplement: SUPPLEMENTAL FILE 1 — Supplemental material. Download spectrum.01175-21-s001.pdf, PDF file, 1.5 MB [file spectrum.01175-21-s001.pdf]

Table of Contents

I. Chemistry: Materials and Methods	*pg. 2*
II. Chemistry: Experimental Procedures	*pg. 3*
III. ^{1}H and ^{13}C NMR Data of the Products	*pg. 16*
V. Microbiology: Materials and Methods	*pg. 25*
VI. Microbiology supplemental figures	*pg. 26*
VI. References	*pg. 29*

I. Chemistry: Materials and Methods

All commercial chemicals were obtained from either Sigma-Aldrich or Fluka and used without further purification. Deuterated solvents for NMR spectroscopy use were purchased from Apollo. Column chromatography was performed using Sigma-Aldrich silica gel 100-200 mesh. Solvents for synthesis purposes were used at GPR grade. Analytical TLC was performed using either Merck Kieselgel 60 F254 silica gel plates or Polygram Alox N/UV254 aluminum oxide plates. Visualization was by UV light (254 nm). NMR spectra were recorded on Bruker DPX-400 Avance spectrometers, operating at 400.13 and 600.1 MHz for ^{1}H NMR; 100.6 and 150.9 MHz for ^{13}C NMR. Shifts are referenced to the internal solvent signals.[1] NMR data were processed using BrukerTOPSPIN software. HRMS spectra were measured on a MicromassLCT electrospray TOF instrument with a WATERS 2690 autosampler and methanol/acetonitrile as carrier solvent. Melting points were determined using a Stuart SP10 melting point apparatus and are uncorrected. Infrared spectra were recorded on a PerkinElmer Spectrum One FT-IR spectrometer equipped with a Universal ATR sampling accessory.

II. Chemistry: Experimental Procedures

The first series of compounds with an amide moiety connecting the diaromatic core to an alloxan-like system (compounds **1** to **3**) were synthesized as shown in Scheme 1.

Reaction between the 4,4′-diaminodiphenyl sulphide with di-*tert*-butyl dicarbonate in the presence of triethylamine afforded the key intermediate **14**, which was condensed with the appropriate acid in the presence of 1-hydroxybenzotriazole, *N,N′*-di-isopropylethylamine and 1-ethyl-3-(3-dimethylaminopropyl)carbodiimide to produce compounds **15** and **3**. Finally, Boc deprotection was achieved upon treatment of compounds **15** and **3**, first with TFA in dichloromethane, leading to the formation of compounds **1** and **2**.

Preparation of derivatives containing a spiro function was next explored and thus, compound **5** was prepared as shown in Scheme 2. Thus, key intermediate **14** was subjected to a Mannich reaction in the presence of barbituric acid and formaldehyde solution (37% wt. in water) to afford compound **4**, which was Boc-deprotected upon treatment with a 1.25 M solution of HCl in methanol yielding compound **5**.

Finally, (thio)urea derivatives **6** and **7** were synthesised. In order to prepare compound **6**, dapsone was reacted with dodecyl isocyanate (Scheme 3). A key intermediate in the formation of the final thiourea containing compound **7** is the isothiocyanate **16** which was prepared by reacting compound **14** with a 0.1 M solution of 1,1'-thiocarbonyldiimidazole in dichloromethane (Scheme 3). Next, **16** was reacted with *mono*-Boc-protected ethylene diamine in dichloromethane for 24 hours yielding the di-

Boc-protected product **17** which was deprotected using a 4 M solution of HCl in 1,4-dioxane to yield the corresponding hydrochloride **7** (Scheme 3).

Scheme 1

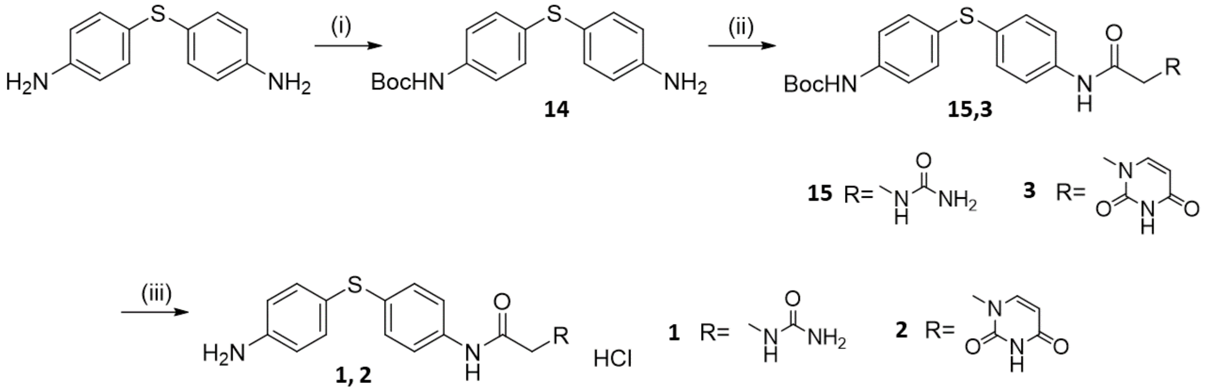

Reagents and conditions. (i) $(Boc)_2O$, TEA, dichloromethane, rt, 16 h, 70%; (ii) 3-ureidopropionic acid (for compound **15**), 3-(2,4-dioxo-3,4-dihydropyrimidin-1(2H)-yl)propanoic acid (for compound **3**), HOBt, DIPEA, EDC, dimethylformamide/dichloromethane, rt, o/n, 75%; (iii) TFA, dichloromethane, rt, 5 h, 85%.

Scheme 2

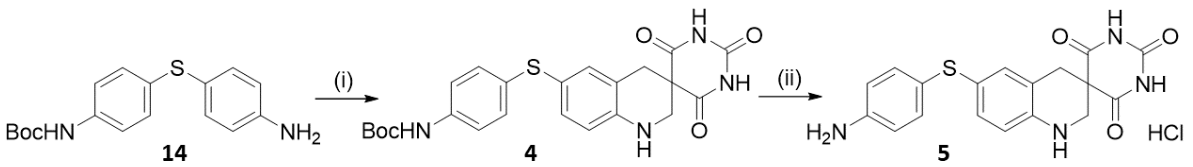

Reagents and conditions. (i) Formaldehyde solution 37% wt. in water, barbituric acid, methanol, reflux, 5 h, 45-50%; (ii) 1.25 M HCl in methanol, methanol, 60 °C, 80%.

Scheme 3

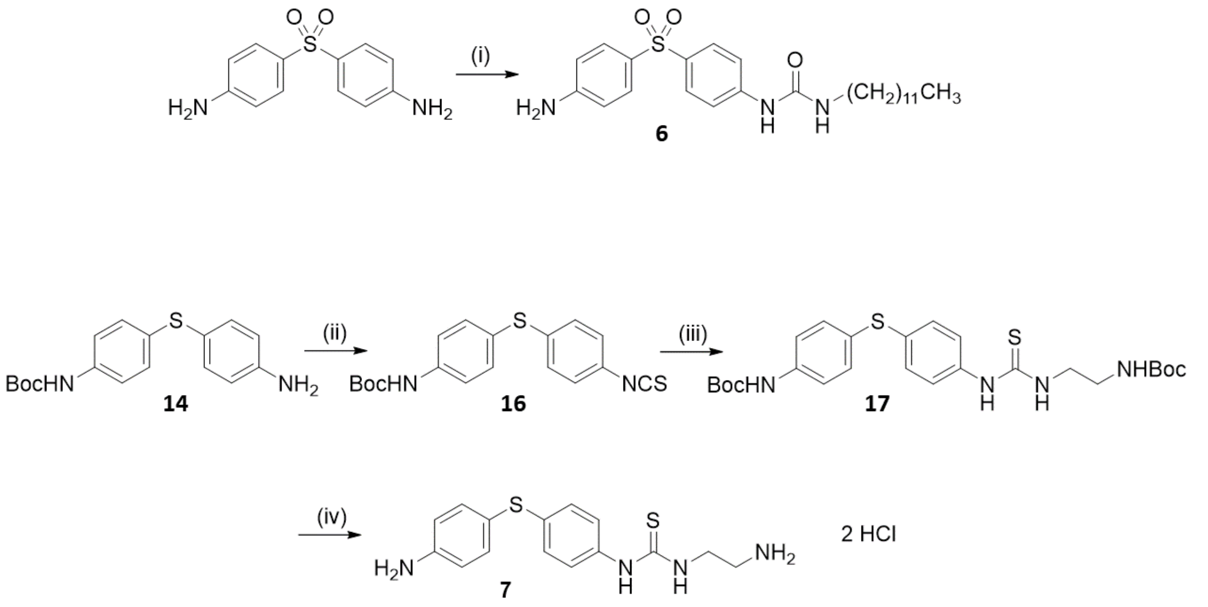

Reagents and conditions. (i) Dodecamethylisocyanate, 1,4-dioxane, reflux, 6 h, r.t., 12%; (ii) 1,1'-thiocarbonyldiimidazole 0.1 M in CH_2Cl_2, 6 h, r.t., 95%; (iii) *mono*-Boc-ethylenediamine, dichloromethane, 24 h, 40 °C, 70%; (iv) 1.25 M HCl in 1,4-dioxane, 4 h, r.t., 59%.

tert-Butyl (4-((4-aminophenyl)thio)phenyl)carbamate (14).

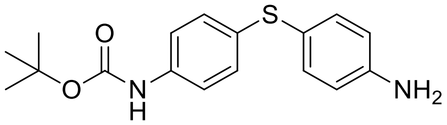

To a stirring solution of (Boc)2O (1.34 g, 6.17 mmol) in dichloromethane (40 mL), 4,4'-diaminodiphenyl sulphide (2.64 g, 12.34 mmol) and Et3N (0.86 mL, 6.17 mmol) were added at 0 °C. The reaction mixture was stirred at the same temperature for 1 h and then was allowed to warm to room temperature and stirred for 16 h. The mixture was washed with brine (2 x 10 mL), dried over Na_2SO_4, filtered and concentrated in vacuo. The resulting crude material was purified by column chromatography over silica gel (hexane/EtOAc, 7:3) to give compound 14 (1.36 g, 70%). ^{1}H-NMR (400 MHz, DMSO-d6) δ 9.30 (s, 1H), 7.33 (d, J = 8.7 Hz, 2H), 7.08 (d, J = 8.5 Hz, 2H), 7.00 (d, J = 8.7 Hz, 2H), 6.55 (d, J = 8.6 Hz, 2H), 5.38 (s, 2H), 1.43 (s, 9H). ^{13}C-NMR (100 MHz, DMSO-d6) δ 153.12 (1C) 149.81 (1C), 138.06 (1C), 135.47(2C), 131.82 (1C), 128.91 (2C), 119.30 (2C), 117.42 (1C), 115.10 (2C), 79.49 (1C), 28.54 (3C). HRMS (N APCI) : m/z 315.1166 [M – 1].

tert-Butyl (4-((4-(3-ureidopropanamido)phenyl)thio)phenyl)carbamate (15).

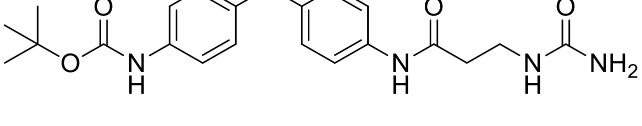

To a solution of commercially available 3-ureidopropionic acid (158 mg, 1.12 mmol) in dimethylformamide (3 mL), 1-hydroxybenzotriazole hydrate (199 mg, 1.3 mmol), N-(3-dimethylaminopropyl)-N'-ethylcarbodiimide hydrochloride (249 mg, 1.3 mmol) and DIPEA (0.383 mL, 2.2 mmol) were added and the reaction mixture was cooled

down to 0 °C and stirred for 15 min. A solution of compound 14 in dichloromethane (3 mL) was added drop by drop at the same temperature and the resulting mixture was stirred at room temperature for 16 h. The reaction mixture was concentrated in vacuo and the residue was taken up in EtOAc (30 mL) and washed with water (2 x 5 mL) and brine (5 mL), dried over Na_2SO_4, filtered and concentrated in vacuo. The crude material was purified by column chromatography over silica gel (dichloromethane/methanol 9:1) to give compound 15 (320 mg, 75%). ^{1}H-NMR (600 MHz, DMSO-d6) δ 10.04 (s, 1H), 9,47 (s, 1H), 7.59 (d, J = 8.7 Hz, 2H), 7.46 (d, J = 8.5 Hz, 2H), 7.24 (d, J = 8.7 Hz, 2H), 7,20 (m, 1H), 6.00 (t, J = 5.7 Hz, 1H), 5,47 (s, 2H), 3.25 (q, J = 6.3 Hz, 2H), 2.44 (t, J = 6.4 Hz, 2H), 1,47 (s, 9H). ^{13}C-NMR (100 MHz, DMSO-d6) δ 170.05 (1C), 158.61 (1C), 152.64 (1C), 139.23 (1C), 138.32 (1C), 132.12 (2C), 130.72 (2C), 129.39 (1C), 126.97 (1C), 119.88 (2C), 118.99 (2C), 79.26 (1C), 40.03 (1C), 37.31 (1C), 28.06 (3C). HRMS (APCI): m/z 431.1754 [M + 1].

tert-Butyl (4-((4-(3-(2,4-dioxo-3,4 dihydropyrimidin1(2H)yl)propanamido)phenyl)thio)-phenyl)carbamate (3).

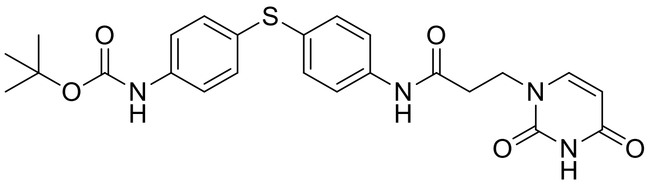

To a solution of commercially available 3-(2,4-dioxo-3,4-dihydropyrimidin-1(2H)-yl)propanoic acid (220 mg, 1.2 mmol) in dimethylformamide/dichloromethane (3 mL/3 mL), 1-hydroxybenzotriazole hydrate (199 mg, 1.3 mmol), 1-ethyl-3-(3-dimethylaminopropyl)carbodiimide hydr*ochloride (249 mg, 1.3 mmol) and DIPEA (0.383 mL, 2.2 mmol) were added and the reaction mixture was cooled down to 0 °C and stirred for 15 min. A solution of compound 14 in dichloromethane (3 mL) was*

added drop by drop at the same temperature and the resulting mixture was stirred at room temperature for 16 h. The reaction mixture was concentrated in vacuo and the residue was taken up in EtOAc (30 mL) and washed with water (2 x 5 mL) and brine (5 mL), dried over Na_2SO_4, filtered and concentrated in vacuo. The crude material was purified by column chromatography over silica gel (dichloromethane/methanol) to give compound 3 (350 mg, 73%).

^{1}H-NMR (600 MHz, DMSO-d6) δ 11.22 (s, 1H), 10.09 (s, 1H), 9.46 (s, 1H), 7.56 (d, J = 7.9 Hz, 1H), 7.51 (d, J = 8.7 Hz, 2H), 7.44 (d, J = 8.6 Hz, 2H), 7.23 (d, J = 8.7 Hz, 2H), 7.17 (d, J = 8.7 Hz, 2H), 5.48 (d, J = 7.8 Hz, 1H), 3.91 (t, J = 6.5 Hz, 2H), 2.68 (t, J = 6.5 Hz, 2H), 1.45 (s, 9H). HRMS (APCI): m/z 483.1697 [M + 1].

N-(4-((4-Aminophenyl)thio)phenyl)-3-ureidopropanamide (1).

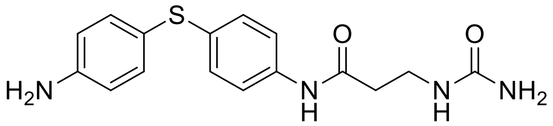

To a stirring solution of compound 15 (107 mg, 0.25 mmol) in dry dichloromethane (4 mL), TFA (0.185 mL, 2.5 mmol) was added slowly at room temperature and the reaction mixture was stirred for 4 h. The mixture was diluted with dichlomethane (20 mL) and washed with a saturated solution of sodium bicarbonate (2 x 5 mL), brine (5 mL), dried over Na_2SO_4, filtered and concentrated in vacuo. The crude material was purified by column chromatography over silica gel (dichloromethane/methanol/NH_3-methanol, 9:1:0.2) to give compound 1 (70 mg, 85%). ^{1}H-NMR (600 MHz, DMSO-d6) δ 9.95 (s, 1H), 7.51 (d, J = 8.6 Hz, 2H), 7.13 (d, J = 8.4 Hz, 2H), 7.03 (d, J = 8.6 Hz, 2H), 6.58 (d, J = 8.4 Hz, 2H), 5.99 (t, J = 5.7 Hz, 1H), 5.46 (bs, 2H), 5.44 (bs, 2H), 3.23 (q, J = 6.3 Hz, 2H), 2.42 (t, J = 6.4 Hz, 2H). ^{13}C-NMR (100 MHz, DMSO-d6) δ 170.35 (1C), 159.10 (1C), 150.02 (1C), 137.58 (1C), 135.89 (2C), 133.36 (1C),

128.31 (2C), 120.24 (2C), 116.80 (1C), 115.19 (2C), 40.52 (1C), 37.74 (1C). HRMS (APCI): m/z 331.1225 [M + 1].

N-(4-((4-Aminophenyl)thio)phenyl)-3-(2,4-dioxo-3,4-dihydropyrimidin-1(2H)-yl)propanamide (2).

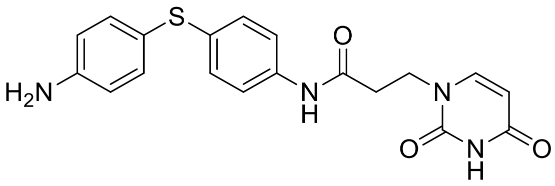

To a stirring solution of compound 3 (100 mg, 0.2 mmol) in dry dichloromethane (4 mL), TFA (0.148 mL, 2 mmol) was added slowly at room temperature and the reaction mixture was stirred for 4 h. The mixture was diluted with dichlomethane (20 mL) and washed with a saturated solution of sodium bicarbonate (2 x 5 mL), brine (5 mL), dried over Na_2SO_4, filtered and concentrated in vacuo. The crude material was purified by column chromatography over silica gel (dichloromethane/ methanol/NH_3-methanol, 9:1:0.2) to give compound 5 (65 mg, 86%). ^{1}H-NMR (600 MHz, DMSO-d6) δ 11.22 (s, 1H), 10.00 (s, 1H), 7.54 (d, J = 7.8 Hz, 1H), 7.42 (d, J = 8.5 Hz, 2H), 7.11 (d, J = 8.3 Hz, 2H), 6.99 (d, J = 8.5 Hz, 2H), 6.56 (d, J = 8.4 Hz, 2H), 5.47 (m, 3H), 3.89 (t, J = 6.3 Hz, 2H), 2.65 (t, J = 6.3 Hz, 2H). ^{13}C-NMR (100 MHz, DMSO-d6) δ 169.00 (1C), 164.19 (1C), 151.27 (1C), 149.99 (1C), 146.63 (1C), 137.13 (1C), 135.95 (2C), 133.76 (1C), 128.17 (2C), 120.38 (2C), 116.61 (1C), 115.18 (2C), 100.96 (1C), 45.01 (1C), 35.51 (1C). HRMS (ESI): m/z 383.1167 [M + 1].

tert-Butyl (4-((2,4,6-trioxo-1,1',3,4,4',6-hexahydro-2H,2'H-spiro[pyrimidine-5,3'-quinolin]-6'-yl)thio)phenyl)carbamate (4).

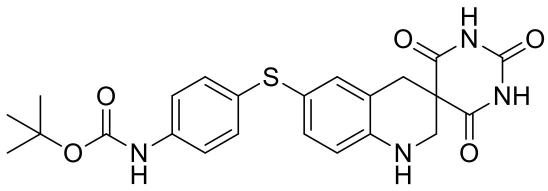

To a stirring solution of compound 14 (100 mg, 0.316 mmol) in methanol (15 mL) were added formalin (0.79 mmol) and barbituric acid (48 mg, 0.379 mmol). The resulting mixture was heated to reflux and stirred for 5 h. The solvent was removed under reduced pressure and the residue was purified by column chromatography over silica gel (dichloromethane/methaol 9:1) to obtain compound 4 (60 mg, 40%). ^{1}H-NMR (600 MHz, DMSO-d6) δ 11.15 (s, 2H), 9.34 (s, 1H), 7.37 (d, J = 8.4 Hz, 2H), 7.07 (m, 3H), 6.97 (dd, J_1 = 8.3 Hz, J_2 = 1.8 Hz, 1H), 6.54 (d, J = 8.3 Hz, 1H), 6.34 (t, J = 3.2 Hz, 1H), 3.39 (d, J = 3.1 Hz, 2H), 3.05 (s, 2H), 1.46 (s, 9H). ^{13}C-NMR (100 MHz, DMSO-d6) δ: 170.96, 152.68 (1C), 150.44 (1C), 143.86 (1C), 137.85 (1C), 133.73 (1C), 131.42 (1C), 130.77 (1C), 129.05 (2C), 120.34 (1C), 118.84 (1C), 118.17 (1C), 114.72 (2C), 79.07 (1C), 47.95 (1C), 47.35 (1C), 30.04 (1C), 28.09 (3C). HRMS (ESI): m/z 467.1409 [M – 1].

6'-((4-Aminophenyl)thio)-1',4'-dihydro-2H,2'H-spiro[pyrimidine-5,3'-quinoline]-2,4,6(1H,3H)-trione (5).

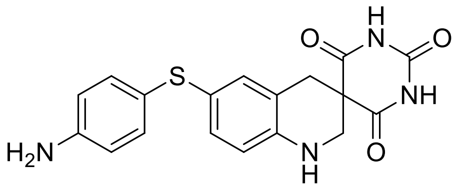

To a stirring solution of compound 4 (40 mg, 0.085 mmol) in methanol (4 mL), 1.25 M HCl in methanol (0.85 mmol) was added dropwise and the resulting solution was heated to 60 °C and stirred for 5 h. The solvent was removed under reduced pressure and the residue obtained was purified by column chromatography over

silica gel (dichloromethane/methanol/NH_3-methanol, 9:1:0.2) to give compound 5 (25 mg, 80%). ^{1}H-NMR (600 MHz,DMSO-d6) δ: 11.06 (s, 2H), 7.02 (d, J = 8.3 Hz, 2H), 6.80 (d, J = 7.5 Hz, 1H), 6.90 (s, 1H), 6.50 (d, J = 8.3 Hz, 2H), 6.45 (d, J = 8.3 Hz, 1H), 6.11 (s, 1H), 5.22 (s, 2H), 3.35 (s, 2H), 2.99 (s, 2H). ^{13}C-NMR (100 MHz, DMSO-d6) δ: 170.90 (2C), 150.44 (1C), 144.2 (1C), 143.86 (1C), 133.73 (1C), 130.9 (1C), 129.08 (2C), 125.9 (1C), 120.34 (1C), 118.84 (1C), 118.17 (1C), 110.72 (2C), 48.02 (1C), 47.39 (1C), 30.1 (1C). HRMS (APCI): m/z 369.1015 [M + 1].

1-(4-((4-Aminophenyl)sulfonyl)phenyl)-3-dodecylurea (6):

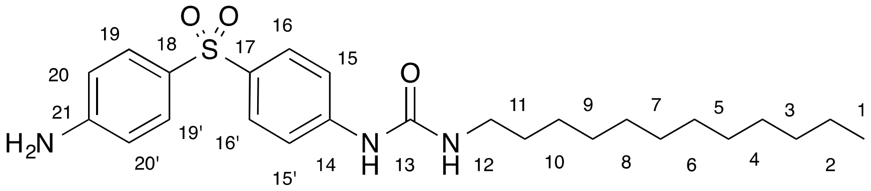

To a solution of dapsone (250 mg, 1 mmol, 1 eq.) in 1,4-dioxane (20 mL) a solution of dodecyl isocyanate (213 mg, 1mmol, 1 eq.) in 1,4-dioxane (1 mL) was added dropwise. The reaction mixture was heated to reflux for 24 hours. The organic layer was washed with brine (2 x 30 mL), dried over $MgSO_4$ and concentrated under vacuum to give a yellow solid. The solid was purified using flash chromatography with silica gel eluting with hexanes:ethyl acetate (33:67) yielding a white solid (12%). **Mp:** 87-90 °C. **v_{max} (cm^{-1}):** 3360 (NH), 2918 (CH), 1672 (CO), 1494 (SO_2), 1283 (C-N). **^{1}H NMR: (400 MHz, DMSO-d_6):** δ 0.85 (t, *J* = 6.9 Hz, 3H, H-1), 1.17 - 1.20 (m, 18H, H-2 - H-10), 1.38 - 1.42 (m, 2H, H-11), 3.03 - 3.07 (m, 2H, H-12), 6.07 (d, 2H, NH_2), 6.27 (t, NH), 6.60 (d, *J* = 8.8 Hz, Ar, 2H, H-20/H-20’), 7.51 – 7.53 (m, 4H, Ar, H-15/H-15’, H-19/H-19’), 7.69 (dd, *J* = 8.9 Hz, Ar, 2H, H-16/H-16’), 8.86 (s, NH).

^{13}C NMR: (150 MHz, DMSO-d_6): δ 13.9 (C-1), 22.0 (C-2), 26.3 (C-3), 28.7-31.3 (C-4 – C-11), 39.1 (C-12), 112.94 (C-20/C-20'), 117.11 (C-15/C-15'), 126.7 (qC, C-18), 127.8 (C-16/C-16'), 129.0 (C-18/C-18'), 134.5 (qC, C-17), 144.5 (qC, C14), 153.2 (qC, C-21), 154.6 (qC, C-13). **HRMS (m/z ES$^-$):** Found 458.2464 (M$^-$ - H. $C_{25}H_{36}N_3O_3S$ Requires 458.2477).

***tert*-Butyl (4-((4-isothiocyanatophenyl)thio)phenyl)carbamate (16)**

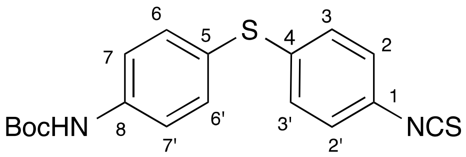

The Boc-protected amine (**14**) (201 mg, 0.63 mmol, 1 eq.) was dissolved in dry CH_2Cl_2 (8 mL) under argon gas. Thiocarbonyldiimidazole (226 mg, 1.26 mmol, 2 eq.) was then added. The reaction mixture was stirred at room temperature for 6 hours. The reaction mixture was then concentrated under vacuum. The crude product was purified by flash chromatography with silica gel eluting at hexanes:ether (70:30) yielding **7** as a white solid (95%). **Mp:** 124 - 126 °C. **ν_{max} (cm^{-1}):** 3311 (NH), 2980 (CH), 2051 (NCS), 1698 (CO). **^{1}H NMR: (400 MHz, DMSO-d_6):** δ 1.48 (d, J = 5.4 Hz, 9H, $(CH_3)_3$), 7.10 - 7.12 (m, 2H, Ar, H-7/H-7'), 7.38 (d, J = 8.5 Hz, 2H, H-2/H-2'), 7.38 - 7.41 (m, 2H, H-6/H-6'), 7.54 (d, J = 7.5 Hz, 2H, H-3/H-3'), 9.60 (s, NH). **^{13}C NMR: (150 MHz, DMSO-d_6):** δ 27.8 $(CH_3)_3$), 79.4 (C((CH_3)), 119.2 (C-7/C-7'), 123.1 (qC), 127.3 (qC), 133.5 (qC), 126.7 (C-2/C-2'), 128.1 (C-3/C-3'), 134.9 (C-6/C-6'), 138.4 (qC), 140.6 (qC), 152.6 (C=O). **HRMS (m/z ES$^-$):** Found 357.0736 (M$^-$ - H. $C_{18}H_{17}N_2O_2S_2$ Requires 357.0731).

***tert*-Butyl (4-((4-(3-(2-((*tert*-butoxycarbonyl)amino)ethyl)thioureido)phenyl)thio)phenyl) carbamate (17)**

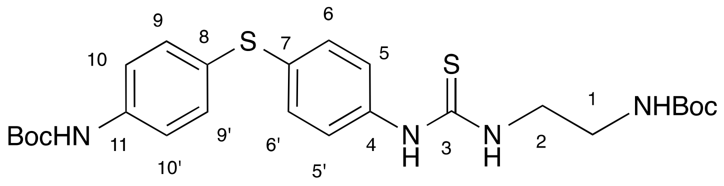

The Boc-protected isothiocyanate **16** (210 mg, 0.59 mmol, 1 eq.) was dissolved in dry CH_2Cl_2 (8 mL). Boc-protected ethylenediamine (94 mg, 0.586 mmol, 1eq.) was dissolved in 2 mL CH_2Cl_2 and added to the isothiocyanate. The reaction was heated to reflux for 22 hours. The solvent was removed under vacuum and the crude product was purified by flash chromatography with silica gel eluting at hexanes:ether, (50:50) yielding a white solid (70%). **Mp:** 88-90 °C. **v_{max} (cm^{-1}):** 3277 (NH), 2976 (CH), 1688 (CO), 1493 (CS). **^{1}H NMR: (600 MHz, DMSO-d_6):** δ 1.35 (s, 9H, $(CH_3)_3$), 1.52 (s, 9H, $(CH_3)_3$), 3.30 – 3.32 (m, 2H, CH_2, H-1), 3.73 (m, 2H, H-2), 4.86 (s, NH), 6.55 (s, NH), 6.85 (s, NH), 7.07 - 7.10 (m, 2H, Ar, H-9/H-9'), 7.16 - 7.20 (m, 2H, Ar, H-6/H-6'), 7.34 - 7.45 (m, 4H, Ar, H-5/H-5', H-10/H-10'), 7.53 (d, NH). **^{13}C NMR: (100 MHz, DMSO-d_6):** δ 28.4 ($(CH_3)_3$), 39.4 (C-1), 46.9 (C-2), 79.8 (<u>C</u>(CH_3)), 81.5, 119.4 (C-5/C-5' or C-10/C-10), 126.1 (C-9/C-9'), 129.6 (C-6/C-6'), 135.0 (C-5/C-5' or C-10/C-10'), 139.3 (qC), 152.6 (qC), 156.6 (qC), 158.3 (qC), 181.44 (qC). **HRMS (m/z ES$^-$):** Found 519.2083 (M^+ + H. $C_{25}H_{35}N_4O_4S_2$ Requires 519.2100)

Dihydrochloride salt of *tert*-butyl (4-((4-(3-(2-((*tert*-butoxycarbonyl)amino)ethyl)thioureido) phenyl)thio)phenyl)carbamate (7)

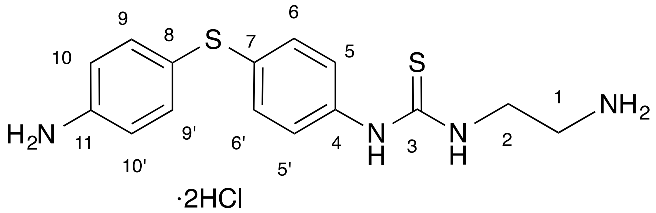

The di-Boc-protected amine **17** (72 mg, 0.15 mmol, 1 eq.) was added to a 25 mL RBF and dissolved in 4 M HCl/dioxane (12 eq.). The reaction mixture was diluted

using a 1:1 solution of CH_2Cl_2/IPA to a final concentration of 0.2 M. The reaction was stirred at room temperature for 4 hours, after which time, a white precipitate formed. The reaction mixture was concentrated under vacuum, dissolved in water and washed with CH_2Cl_2 (4 x 5 mL). The aqueous layer was concentrated under vacuum to yield the dihydrochloride salt **7** as an orange solid (59%). **Mp:** 90-92 ^oC. **v_{max} (cm^{-1}):** 3300 (NH), 2853 (CH), 1543 (CS), 1313. **^1H NMR: (600 MHz, $CDCl_3$):** δ 3.00 - 3.03 (m, 2H, H-1), 3.72 - 3.76 (m, 2H, H-2), 7.23 (m, Ar, 2H, H-6/H-6’), 7.30 - 7.33 (m, Ar, 4H, H-9/H-9’, H-10/H-10’), 7.56 (d, *J* = 8.6 Hz, 2H, H-5/H-5’). **^{13}C NMR: (150 MHz, DMSO):** δ 37.6 (C-1), 40.0 (C-2), 122.5 (C-10/C-10’), 123.6 (C-5, C-5’), 124.1 (qC), 128.5 (qC, C-7), 131.0 (C-9/C-9’), 131.9 (C-6/C-6’), 132.6 (qC), 139.2 (qC, C-4), 180.9 (C-3). **HRMS (m/z ESI^-):** Found 317.0890 (M^- - H. $C_{15}H_{17}N_4S_2$ Requires 317.0895).

III. 1H and 13C NMR Data of the Products

Compound 14

^{1}H-NMR (compound 14)

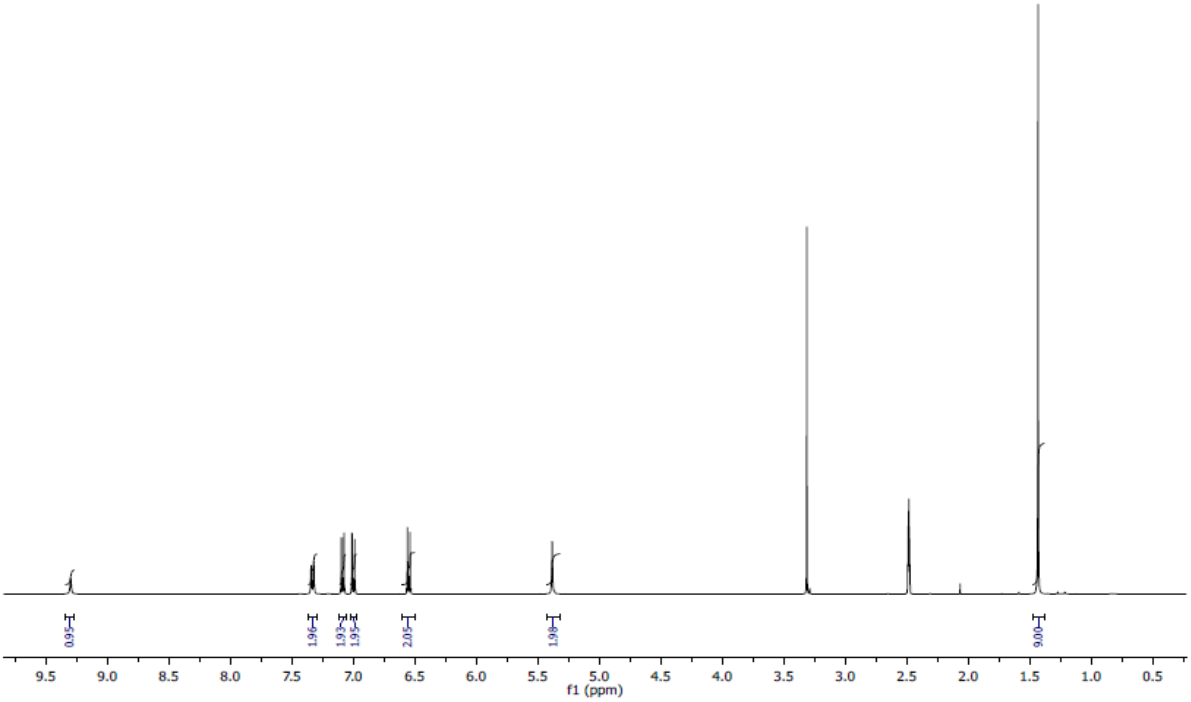

^{13}C-NMR (compound 14)

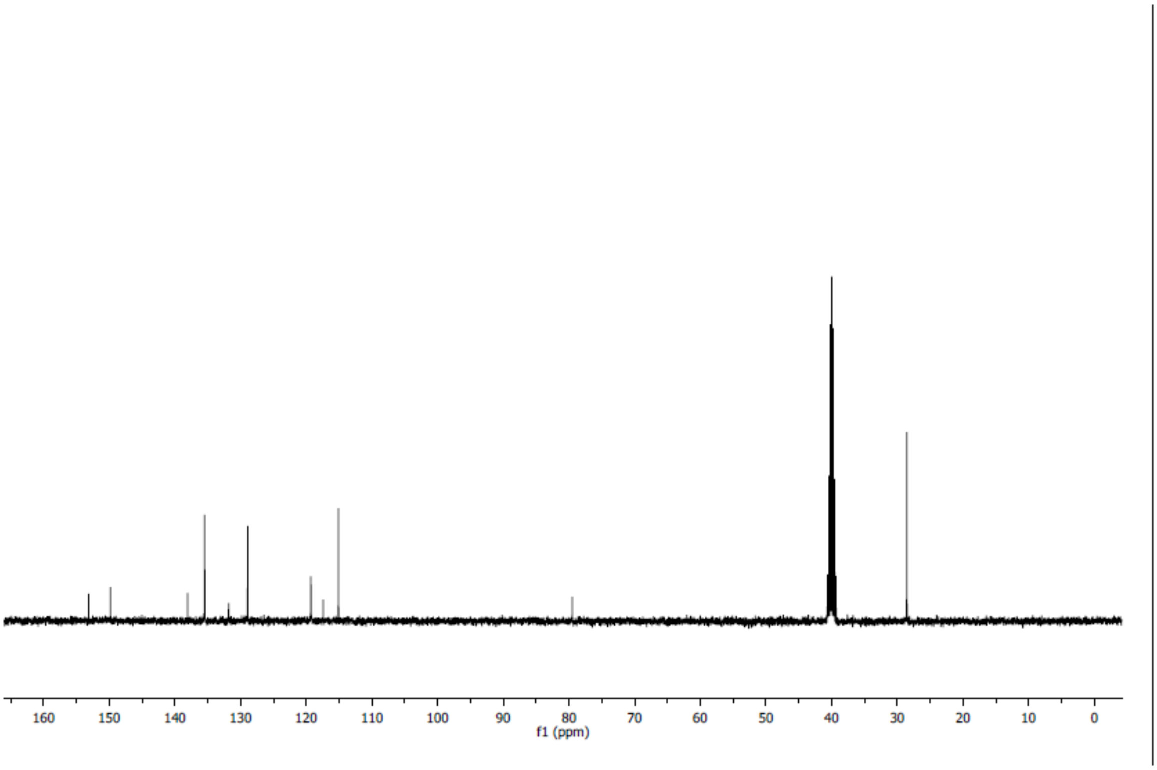

Compound 15

¹H-NMR (compound 15)

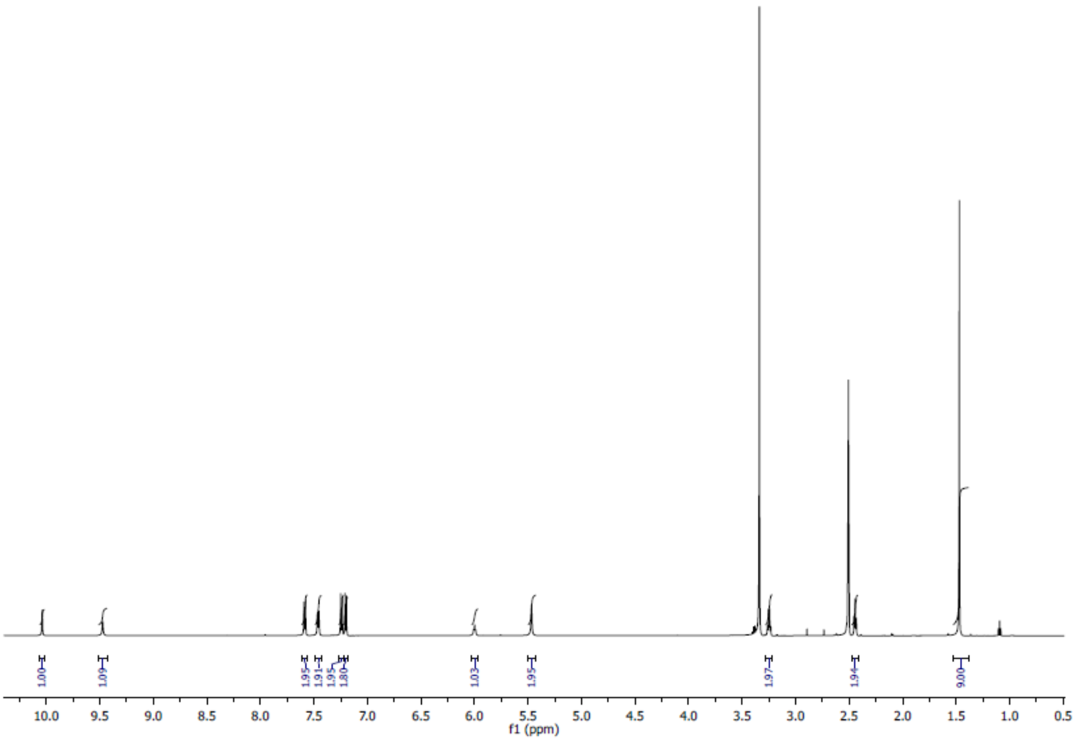

¹³C-NMR (compound 15)

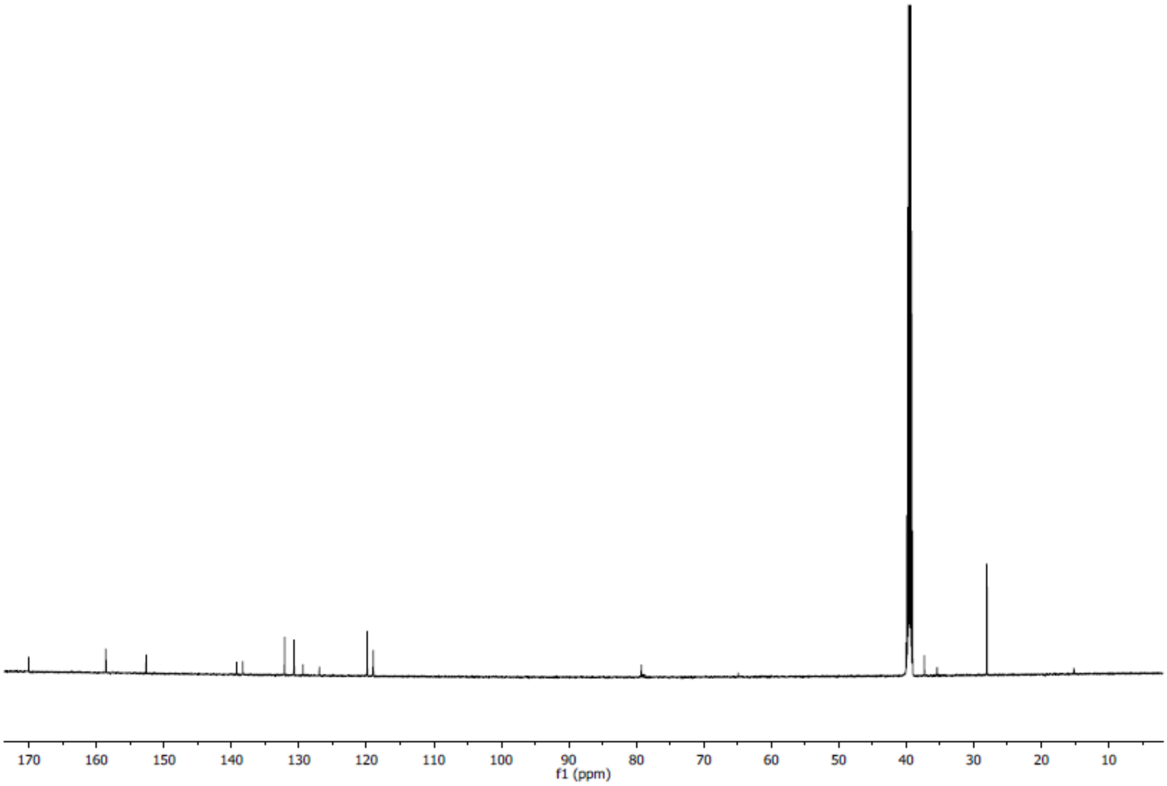

Compound 3

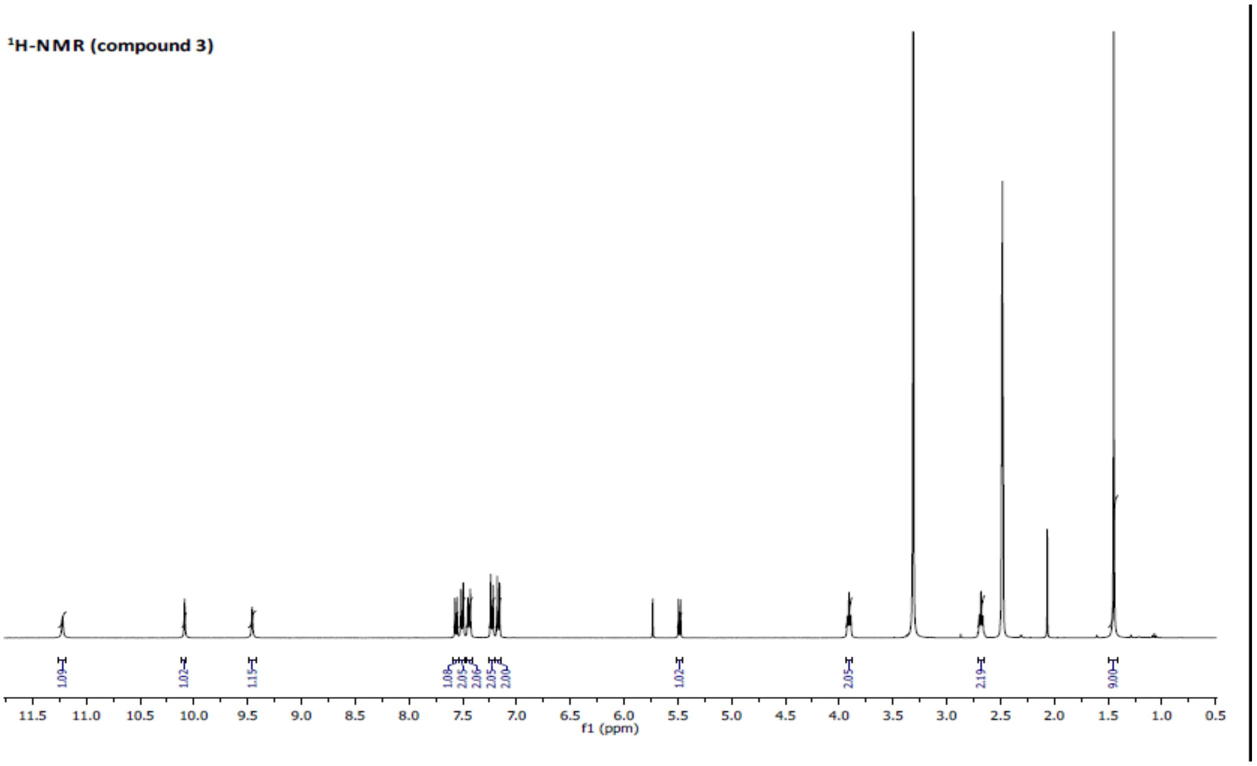

¹H-NMR (compound 3)
1.09
1.02
1.15
1.08
2.05
2.06
2.05
2.00
1.02
2.05
2.19
9.00
11.5 11.0 10.5 10.0 9.5 9.0 8.5 8.0 7.5 7.0 6.5 6.0 5.5 5.0 4.5 4.0 3.5 3.0 2.5 2.0 1.5 1.0 0.5
f1 (ppm)

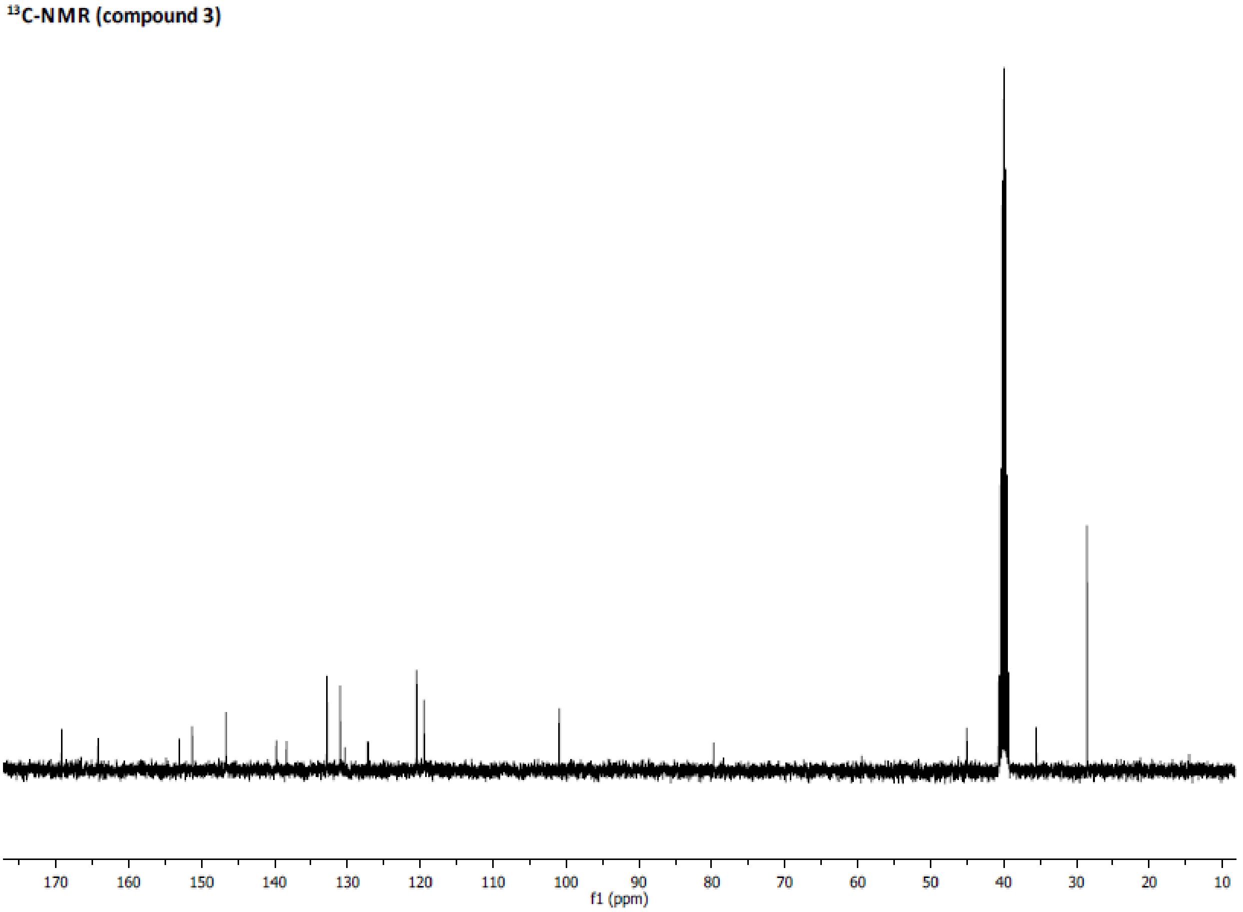

¹³C-NMR (compound 3)
170 160 150 140 130 120 110 100 90 80 70 60 50 40 30 20 10
f1 (ppm)

Compound 2

^{1}H-NMR (compound 2)

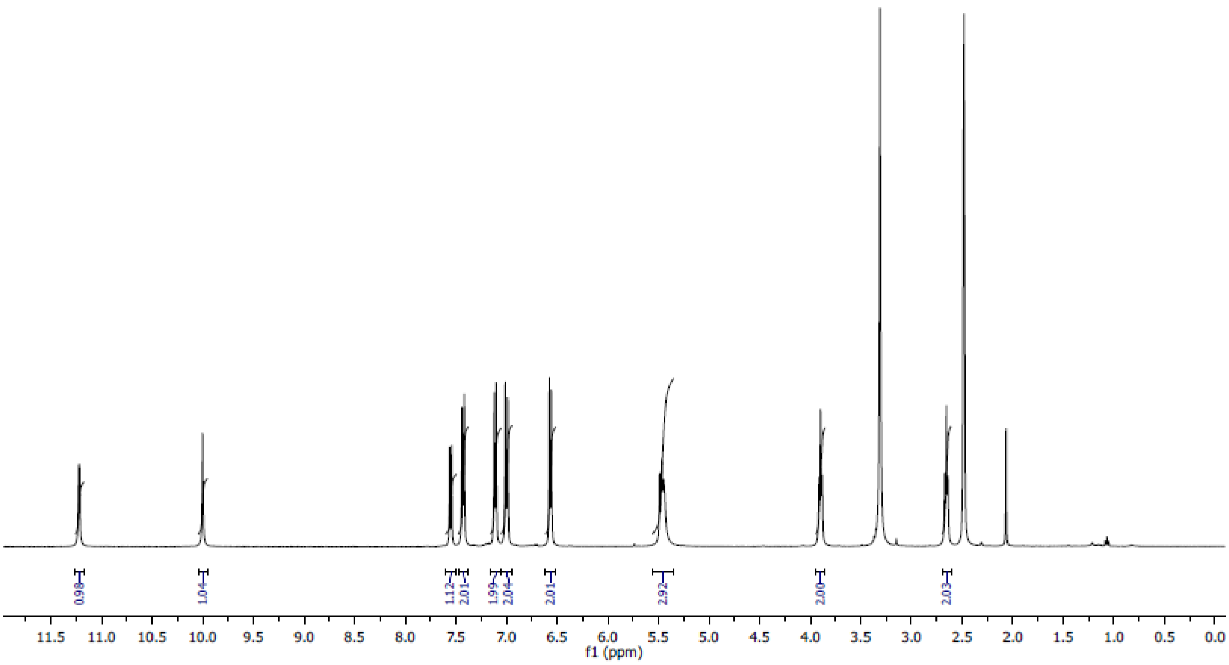

^{13}C-NMR (compound 2)

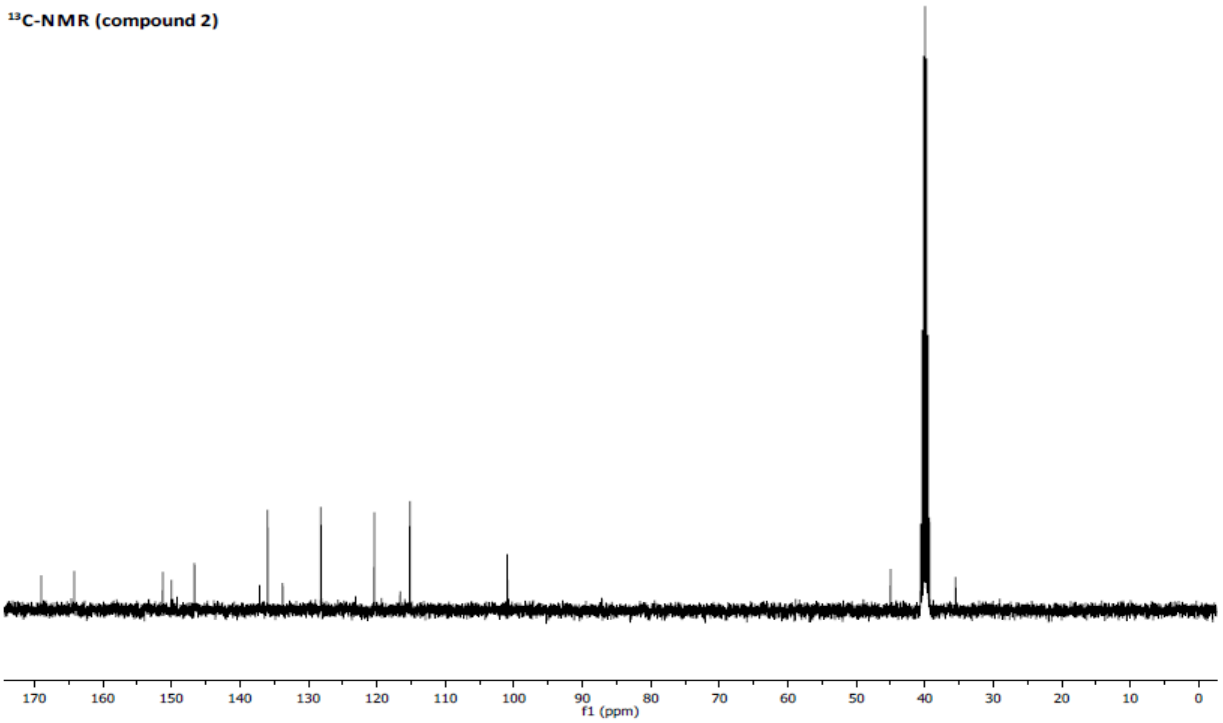

Compound 1

^{1}H-NMR (compound 1)

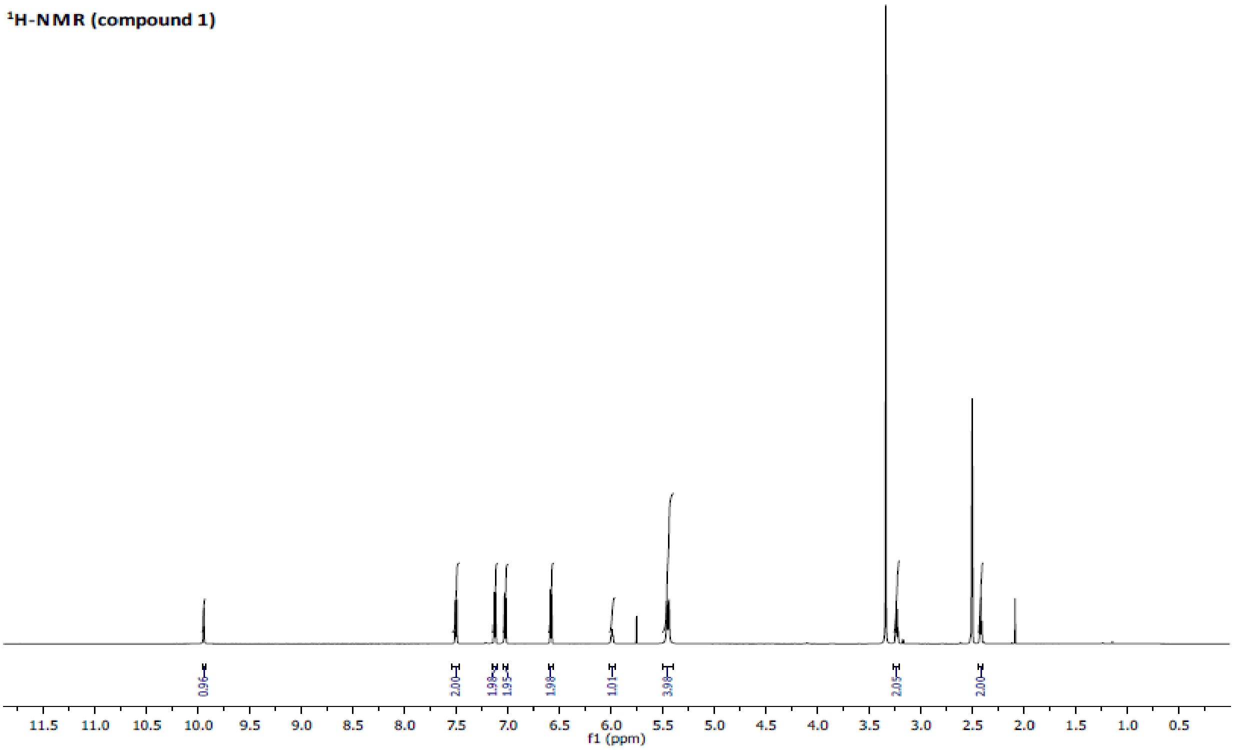

^{13}C-NMR (compound 1)

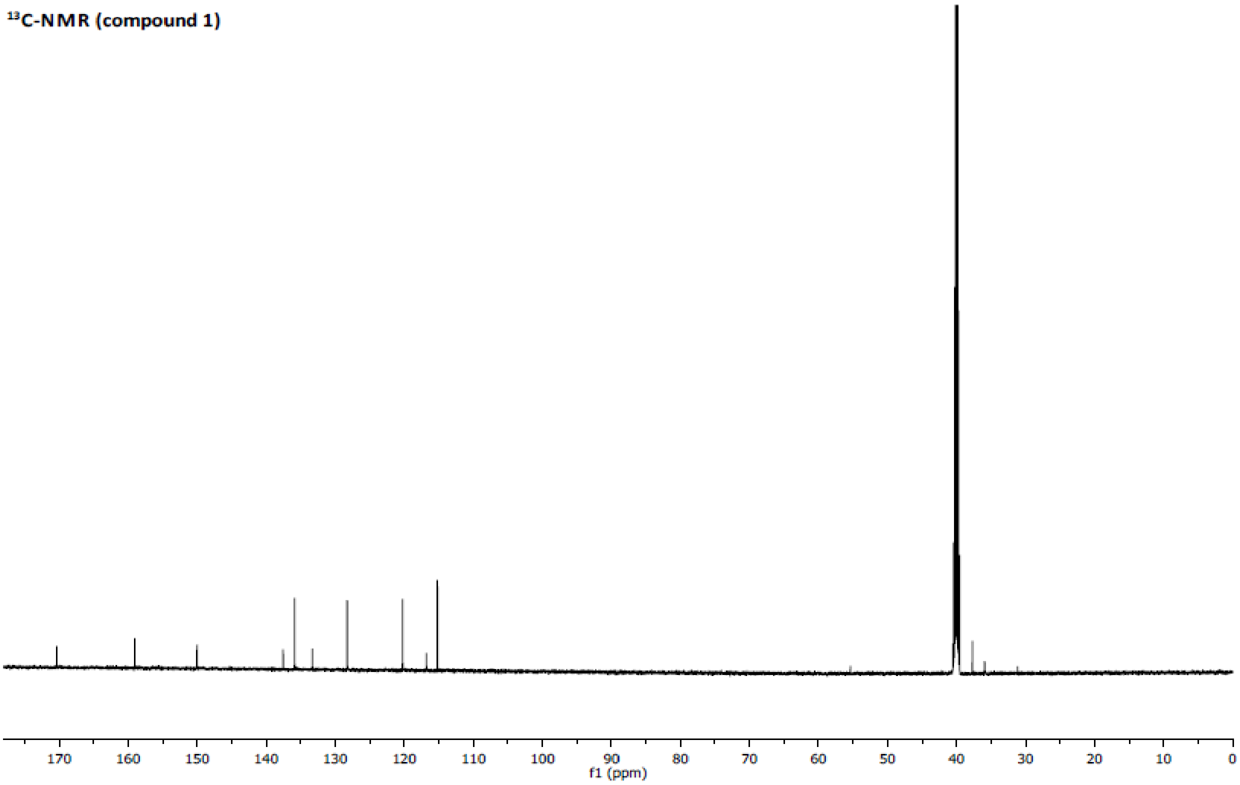

Compound 4

^{1}H-NMR (compound 4)

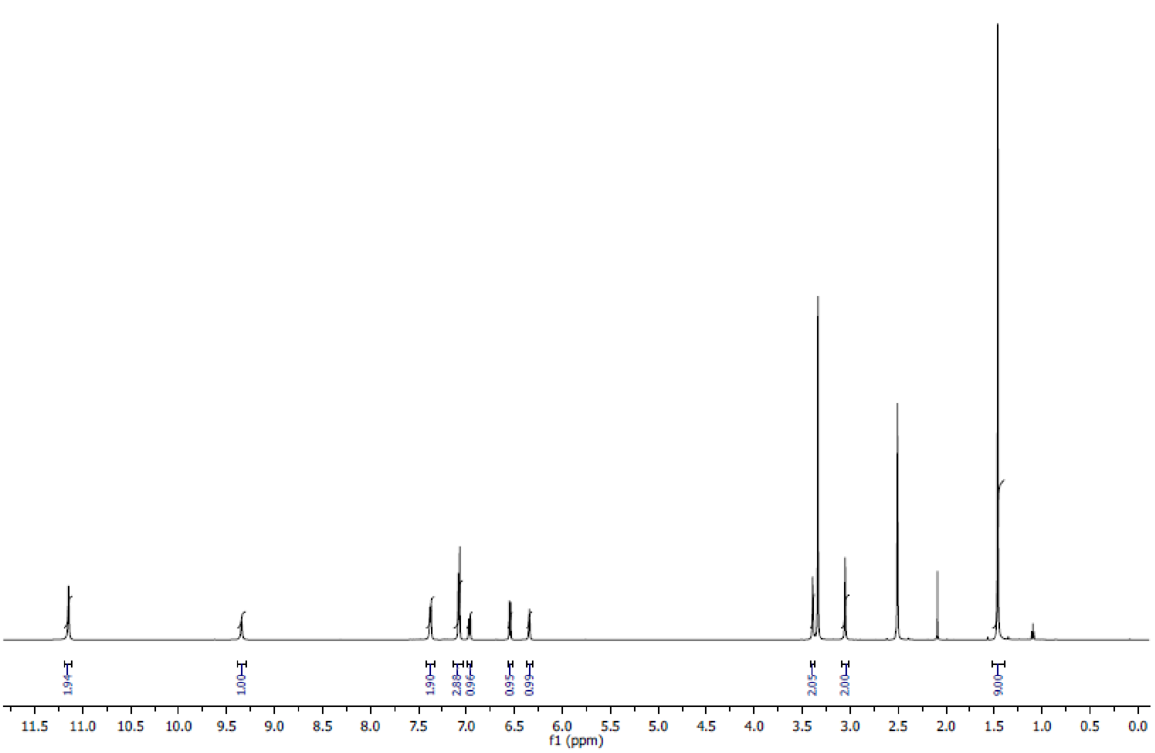

^{13}C-NMR (compound 4)

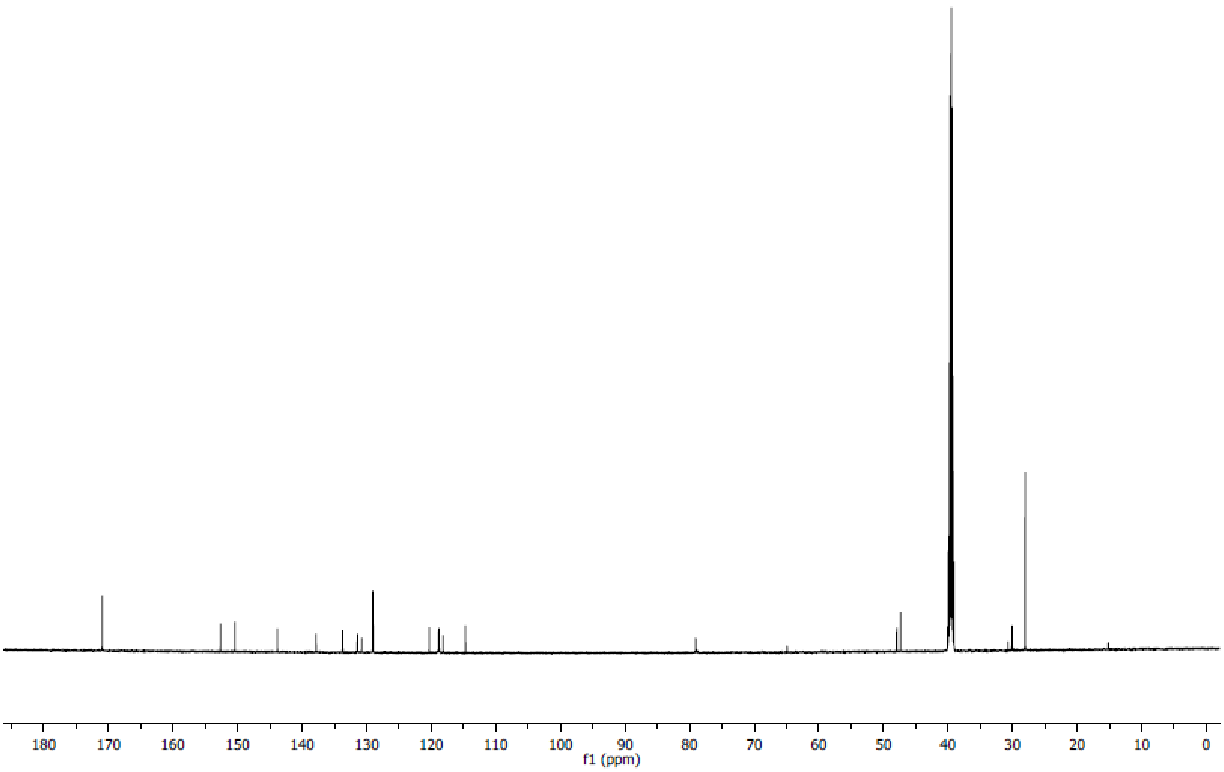

Compound 5

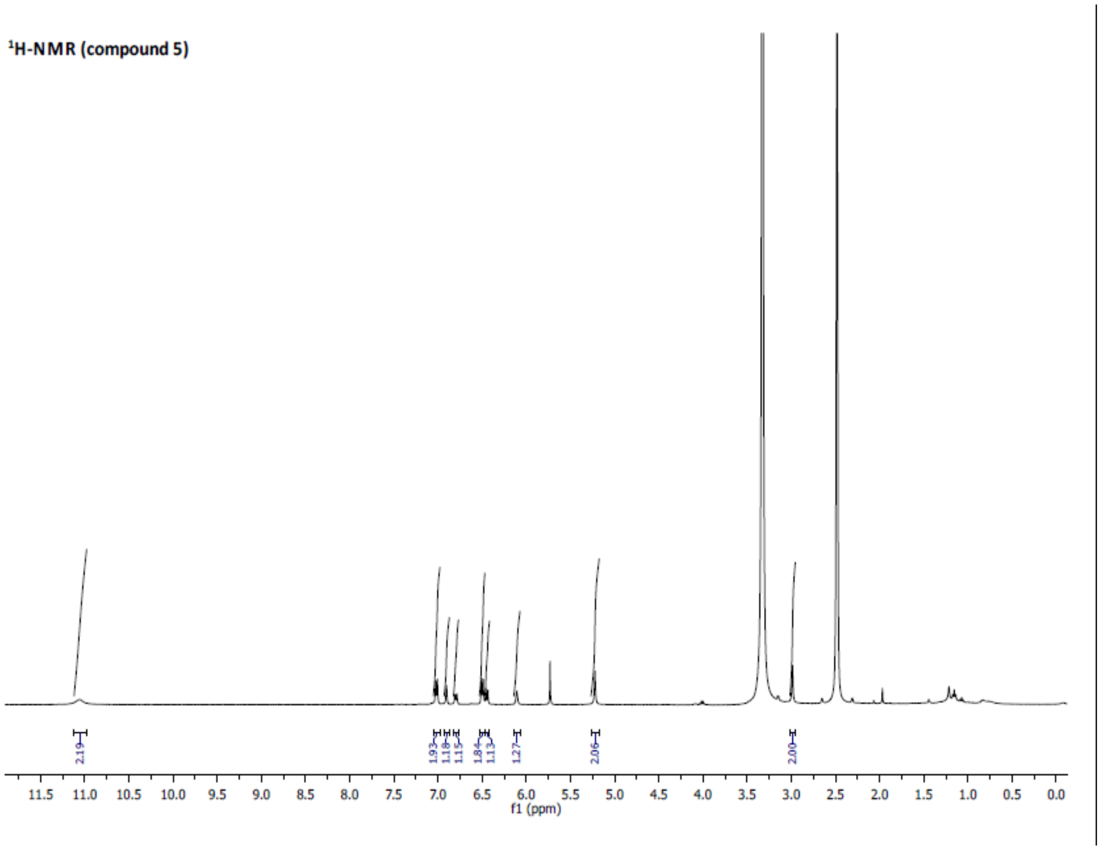

High Resolution Mass Spectroscopy of Compound 5

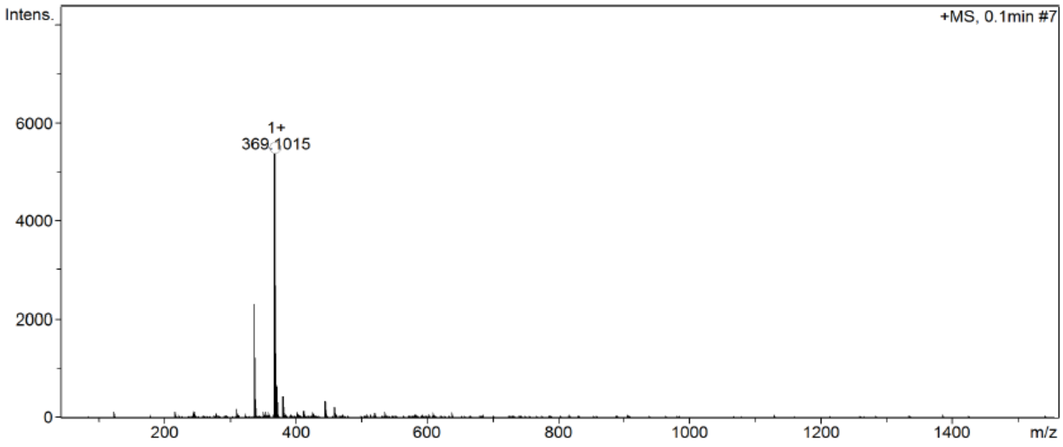

Meas. m/z	#	Ion Formula	m/z	err [mDa]	err [ppm]	rdb	N-Rule	e⁻ Conf	mSigma
369.101484	1	C18H17N4O3S	369.101588	-0.1	-0.3	12.5	ok	even	25.8

Compound 6

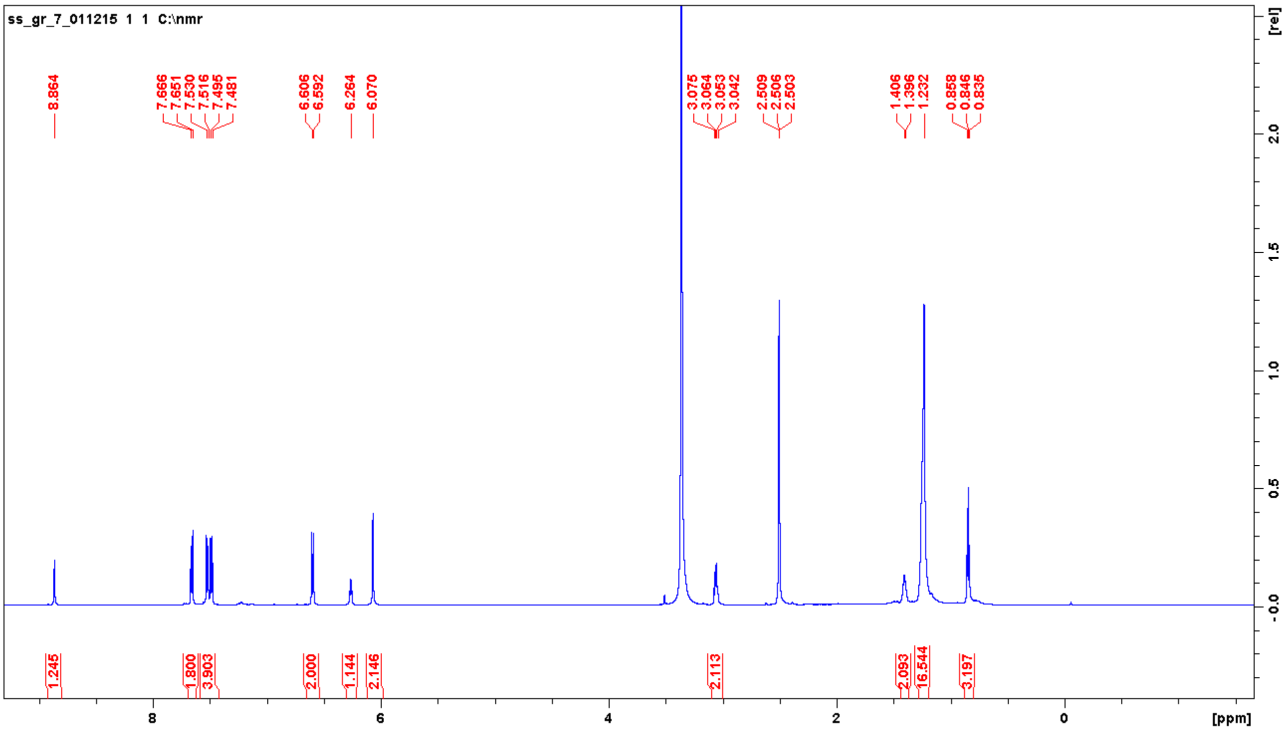
ss_gr_7_011215 1 1 C:\nmr
8.864
7.666
7.651
7.530
7.516
7.495
7.481
6.606
6.592
6.264
6.070
3.075
3.064
3.053
3.042
2.509
2.506
2.503
1.406
1.396
1.232
0.858
0.846
0.835
1.245
1.800
3.903
2.000
1.144
2.146
2.113
2.093
16.544
3.197
[ppm]
[rel]

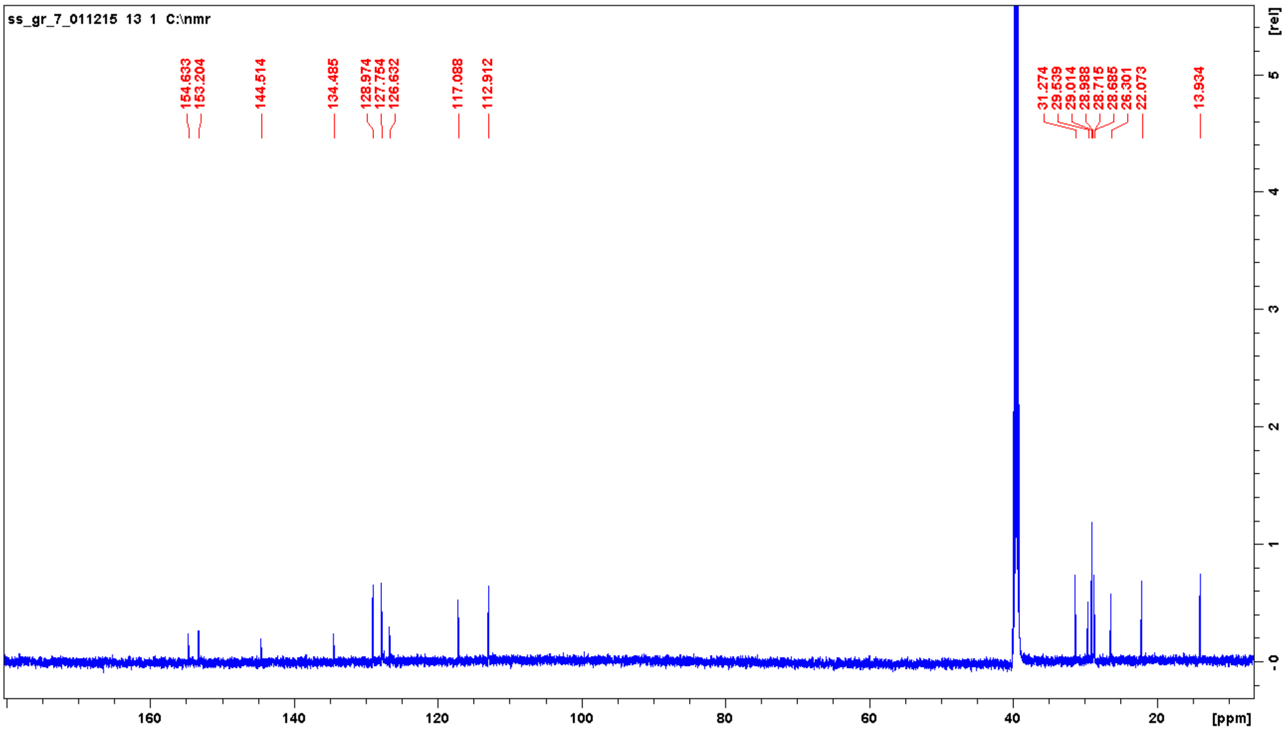
ss_gr_7_011215 13 1 C:\nmr
154.633
153.204
144.514
134.485
128.974
127.754
126.632
117.088
112.912
31.274
29.539
29.014
28.988
28.715
28.685
26.301
22.073
13.934
[ppm]
[rel]

Compound 7

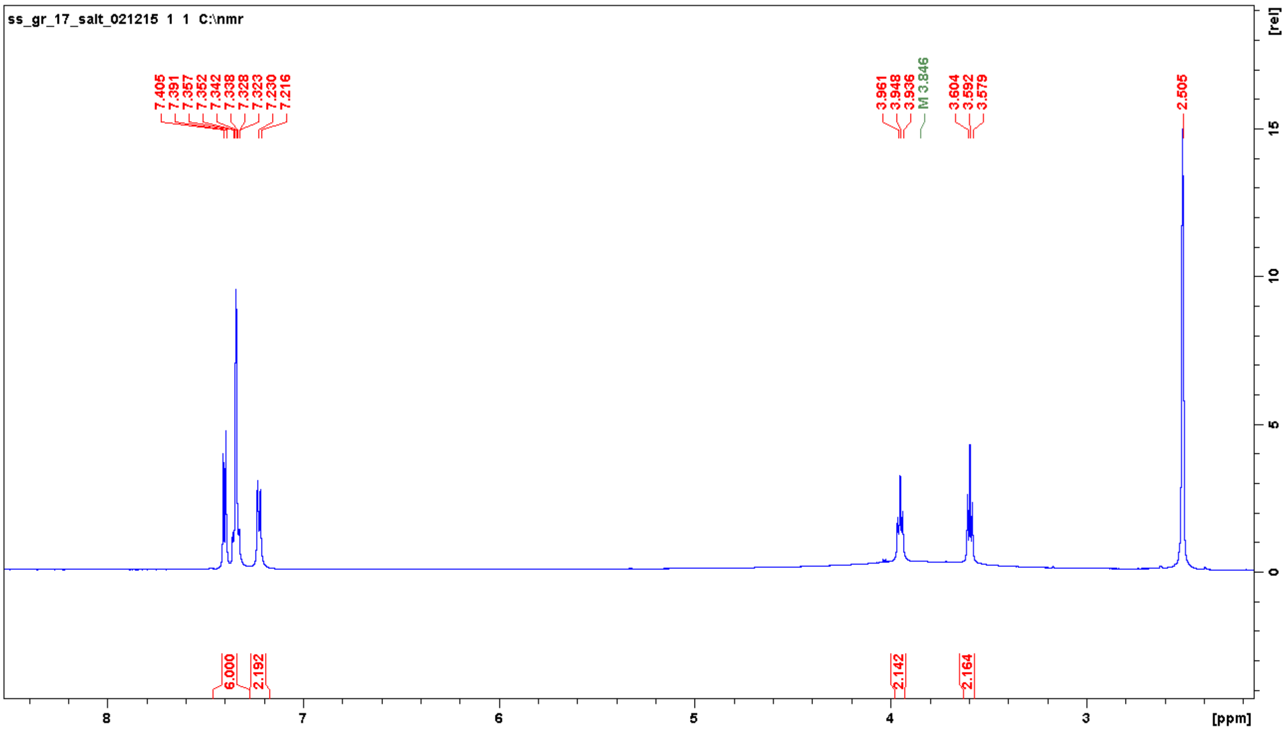

ss_gr_17_salt_021215 1 1 C:\nmr
7.405
7.391
7.357
7.352
7.342
7.338
7.328
7.323
7.230
7.216
3.961
3.948
3.936
M 3.846
3.604
3.592
3.579
2.505
6.000
2.192
2.142
2.164
[rel]
[ppm]

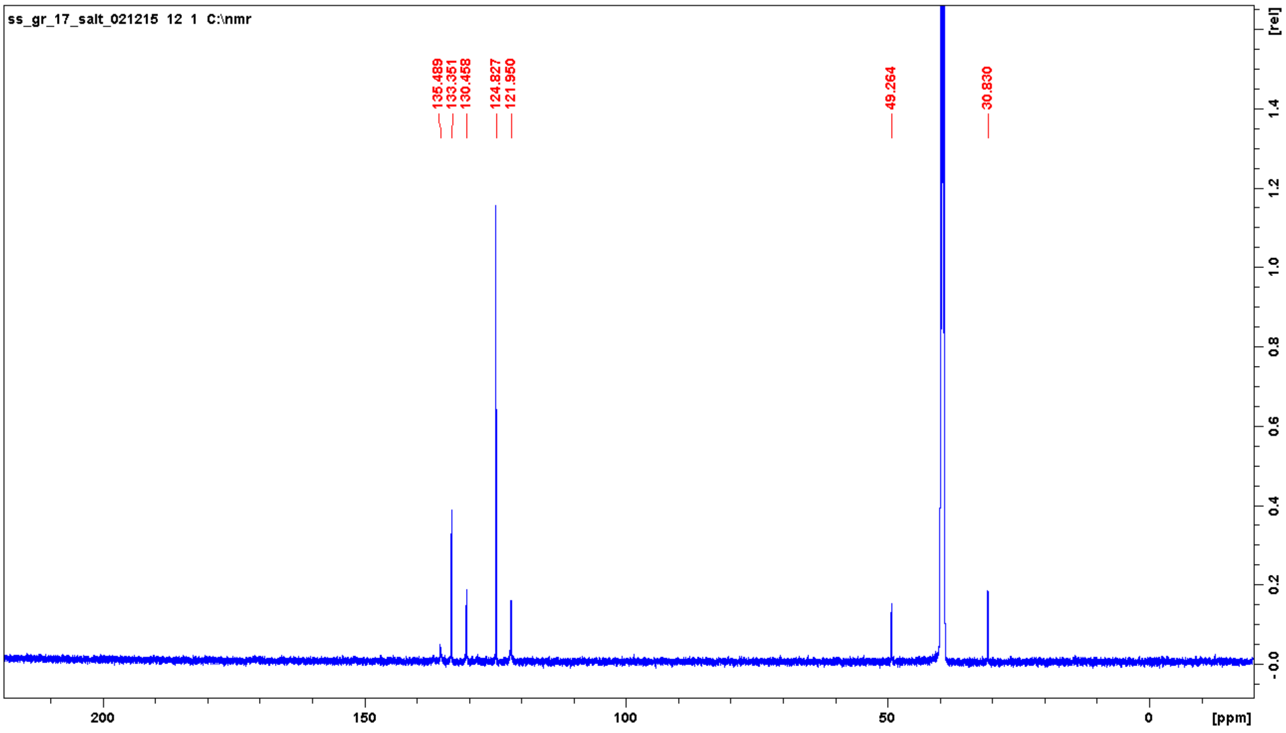

ss_gr_17_salt_021215 12 1 C:\nmr
135.489
133.351
130.458
124.827
121.950
49.264
30.830
[rel]
[ppm]

V. Microbiology: Materials and Methods

Toxicity assay

The MTT toxicity assay (1) (Vybrant MTT Cell Proliferation Assay, Molecular Probes, Or, USA) was used to measure cytotoxicity. Myeloid lineage, U-937 cells (2) were grown in RPMI-1640 (Gibco) culture medium supplemented with 10% foetal calf serum, 1% L-glutamine and 1% Penicillin/Streptomycin (Gibco). U-937 cells were centrifuged and cells diluted with fresh medium containing 1.2 mM MTT solution to a concentration of (5-6 x 10^6 per mL) and incubated at 37 °C for 2 h. Cells were centrifuged and the pellet dissolved in DMSO. The absorbance was read at 490 nm using the Wallac $Victor_2$, (Shelton, Connecticut, USA).

Platelet aggregation

Platelet aggregation was performed in platelet rich plasma according to O'Brien *et al.* (3).

Agglutination assay

S. aureus Newman (stationary phase) was washed and diluted to an OD_{600} of 2.5. This was mixed with 10 mg/mL of fibrinogen in 1:1 ratio resulting in a final concentration 5 mg/mL fibrinogen. Fibrinogen-bacteria aggregates clump and settle at the bottom of the tube resulting in a clear solution. These were compared to the turbid bacterial suspension in buffer by visual inspection.

VI. Microbiology supplemental figures

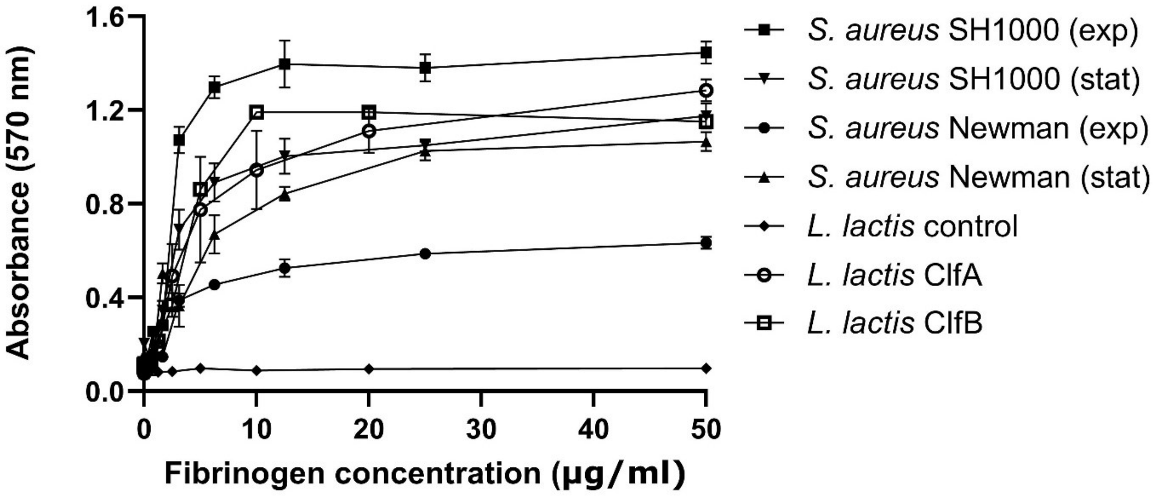

Figure S1: Comparison of *S. aure*us and *L. lactis* strains adhesion to fibrinogen. Fibrinogen was used to coat plates at 4 °C overnight at concentrations from 0-50 µg/mL. Wells were washed with PBS and blocked with 1% BSA for 90 min. Bacterial cells were washed and resuspended to an OD_{600} of 1.0 and added to the wells, incubating for 2 hours at 37 °C. Adherent cells were washed, fixed, stained with crystal violet and wells further washed. The dye was dissolved in acetic acid and the absorbance measured at 570 nm.

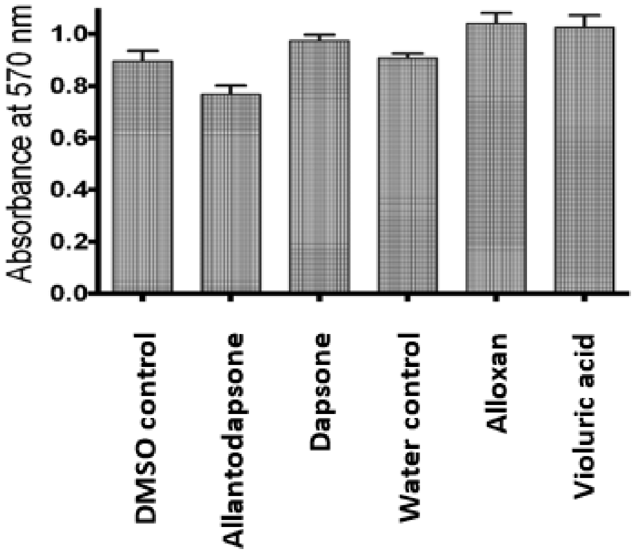

Figure S2: Allantodapsone does not inhibit adhesion of *S. aureus* SH1000 to fibronectin. Fibronectin was used to coat plates at 4 °C overnight at 20 µg/mL. Wells were washed with PBS and blocked with 1% BSA. Washed bacteria (OD_{600} of 1) were added with the relevant concentration of allantodapsone or vehicle control, and incubated for 2 hours at 37 °C. Adherent cells were washed, fixed, stained with crystal violet and wells further washed. The dye was dissolved in acetic acid and the absorbance measured at 570 nm. Statistics were carried out using repeated measures ANOVA followed by Dunnett's post-test using GraphPad Prism (n=3, *p*-value > 0.05).

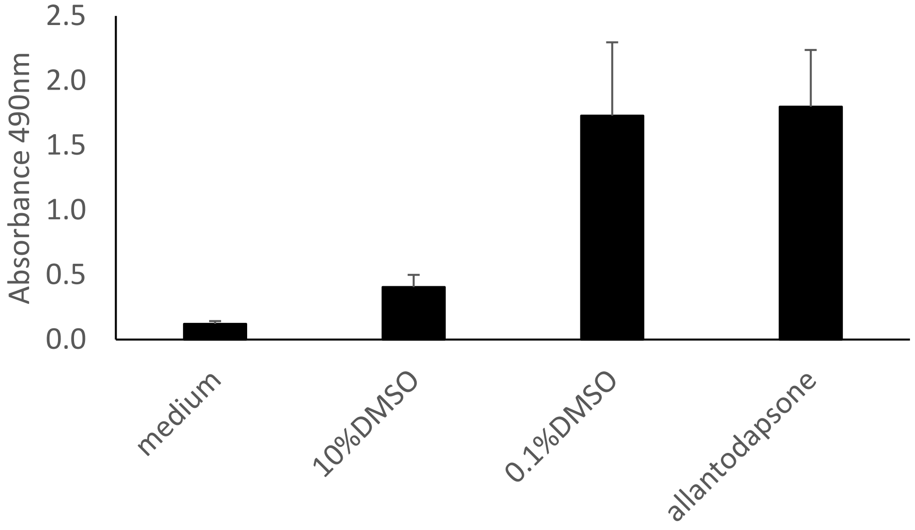

Figure S3: Allantodapsone has no toxic effect on U-937 cells. Allantodapsone was tested for toxicity using the MTT assay (Vybrant^{TM} MTT Cell Proliferation Assay, Molecular Probes, OR, USA). 10% DMSO is used as a positive control in this assay. Allantodapsone was incubated with undifferentiated U-937 monocyte cells at 100 µM. Medium represents the background control due to the media with no cells present (n=2).

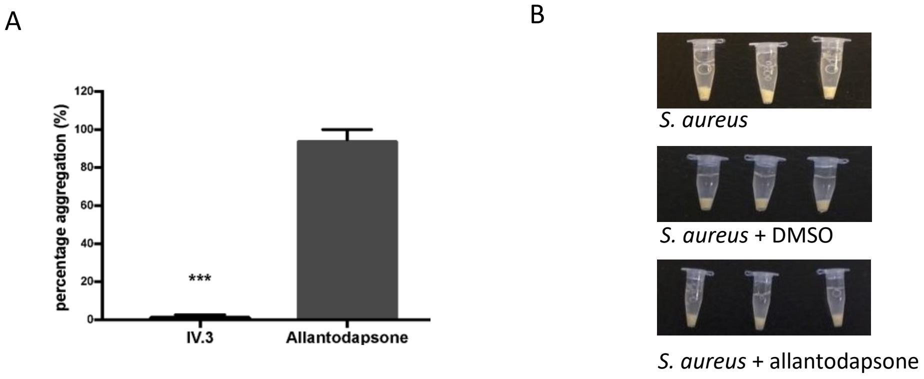

Figure S4: Allantodapsone did not inhibit *S. aureus* Newman induced platelet aggregation or bacterial agglutination. (A) Platelets were incubated with 100 µM allantodapsone or the DMSO vehicle control for 5 minutes prior to aggregation. 10%

of *S. aureus* Newman was used as an agonist to stimulate platelet aggregation. The FcγRIIa antibody IV.3 (StemCell Technologies, London, UK) was used at a concentration of 100 µM. Data was normalized to the DMSO vehicle control. Data was analysed by repeated measures ANOVA followed by a multiple comparison Dunnett's test. *P*-value=NS. Error bars represent SEM values (n= 3). (B) *S. aureus* Newman (stationary phase) was washed and diluted to an OD_{600} of 2.5. This was mixed with 10 mg/mL of fibrinogen in 1:1 ratio resulting in a final concentration 5 mg/mL fibrinogen and 100 µM allantodapsone. Fibrinogen-bacteria aggregates clump and settle at the bottom of the tube resulting in a clear solution.

VI. References

1. Mosmann T. 1983. Rapid colorimetric assay for cellular growth and survival: application to proliferation and cytotoxicity assays. J Immunol Methods 65:55-63.
2. Sundstrom C, Nilsson K. 1976. Establishment and characterization of a human histiocytic lymphoma cell line (U-937). Int J Cancer 17:565-77.
3. O'Brien L, Kerrigan SW, Kaw G, Hogan M, Penades J, Litt D, Fitzgerald DJ, Foster TJ, Cox D. 2002. Multiple mechanisms for the activation of human platelet aggregation by Staphylococcus aureus: roles for the clumping factors ClfA and ClfB, the serine-aspartate repeat protein SdrE and protein A. Mol Microbiol 44:1033-44.
